# Elevation of β-galactoside α2,6-sialyltransferase 1 in a fructose-responsive manner promotes pancreatic cancer metastasis

**DOI:** 10.18632/oncotarget.13845

**Published:** 2016-12-09

**Authors:** Chi-Che Hsieh, Yi-Ming Shyr, Wen-Ying Liao, Tien-Hua Chen, Shin-E Wang, Peir-Chuen Lu, Pei-Yu Lin, Yan-Bo Chen, Wan-Yu Mao, Hsin-Ying Han, Michael Hsiao, Wen-Bin Yang, Wen-Shan Li, Yuh-Pyng Sher, Chia-Ning Shen

**Affiliations:** ^1^ The Ph.D. Program for Cancer Biology and Drug Discovery, China Medical University and Academia Sinica, Taiwan; ^2^ Genomics Research Center and Academia Sinica, Taipei, Taiwan; ^3^ Institute of Chemistry, Academia Sinica, Taipei, Taiwan; ^4^ Department of Surgery, Taipei Veterans General Hospital, Taipei, Taiwan; ^5^ Institute of Anatomy and Cell Biology and National Yang-Ming University, Taipei, Taiwan; ^6^ Department of Biotechnology and Laboratory Science in Medicine, National Yang-Ming University, Taipei, Taiwan; ^7^ Graduate Institute of Life Sciences, National Defense Medical Center, Taipei, Taiwan; ^8^ Graduate Institute of Biomedical Sciences, China Medical University, Taichung, Taiwan; ^9^ Graduate Institute of Clinical Medicine, Taipei Medical University, Taipei, Taiwan; ^10^ Faculty of Medicine, National Yang-Ming University, Taipei, Taiwan

**Keywords:** fructose, pancreatic ductal adenocarcinoma, metastasis, β-galactoside α2, 6-sialyltransferase 1

## Abstract

Pancreatic ductal adenocarcinoma (PDAC) is an aggressive type of pancreatic cancer with clinical characteristics of local invasion and early metastasis. Recent cohort studies indicate high fructose intake is associated with an increase in pancreatic cancer risk. However, the mechanisms by which fructose promotes pancreatic tumorigenesis remain unclear. Herein, Kras^+/LSLG12D^ mice were crossed with Elas-CreER transgenic mice to determine whether fructose intake directly contributes to tumor formation. Orthotopic tumor-xenograft experiments were performed to determine whether fructose substitution enhances the metastatic potential of PDAC cells. The mechanisms underlying the effects of fructose were explored by RNAseq analysis in combination with high-performance anion exchange chromatography. Dietary fructose was initially found to promote the development of aggressive pancreatic cancer in mice conditionally expressing Kras^G12D^ in the adult pancreas. We further revealed that fructose substitution enhanced the metastatic potential of human PDAC cell via selective outgrowth of aggressive ABCG2-positive subpopulations and elevating N-acetylmannosamine levels that upregulated β-galactoside α2,6-sialyltransferase 1 (ST6Gal1), thereby promoting distant metastasis. Finally, we observed that PDAC patients expressing higher levels of ST6Gal1 and GLUT5 presented poorer prognosis compared to other groups. In conclusion, our findings have elucidated a crucial role of ST6Gal1 in regulating the invasiveness of PDACs in a fructose-responsive manner.

## INTRODUCTION

Pancreatic ductal adenocarcinoma (PDAC) is one of the most aggressive cancers in humans [1, 2]. The current prognosis of PDAC remains poor with a median survival of approximately 9 months, and an overall five-year survival rate of 6% (combining all stages) [1, 2]. PDAC is notorious for its tendency toward early metastatic dissemination, and over 80% of patients with PDACs are diagnosed with unresectable advanced disease at the time of diagnosis [2–4], suggesting that the poor survival of patients with pancreatic cancer is likely due to early metastasis. Therefore, identification of mechanisms that govern metastatic dissemination might lead to an improvement in the management of pancreatic cancer metastasis.

Fructose intake has increased by at least 5-fold in the 21st century compared to that in the 19th century [5]. Several cohort studies suggest that patients ingesting high sugar-sweetened beverages or free fructose are at greater risks of developing pancreatic cancer [6–8]. High fructose consumption in humans is associated with the development of metabolic abnormalities such as obesity and insulin resistance [9, 10], which may account for the associated increased risk of pancreatic cancer [11]. However, fructose may directly contribute to tumor formation or passively promote tumorigenesis. In fact, earlier work showed that dietary fructose promotes the generation of acinar-cell tumor nodules in the pancreas of rats treated with N-nitrosomorpholine [12]. Recent work further revealed that PDAC cells can be grown in fructose-containing medium without glucose, suggesting that fructose can serve as an alternate carbohydrate substrate for cancer growth [13]. These findings not only confirm that pancreatic cancer cells can readily metabolize fructose to support their proliferative needs, but also hint at a possible link between fructose and tumorigenesis. Furthermore, glucose transporter 5 (GLUT5), which has a high affinity for fructose [14], is highly expressed in breast cancer [15]. A recent study demonstrated that fructose treatment can promote the migration and invasion of human breast cancer [16].

Aberrant sialylation is often observed in cancer cells [17, 18]. Hypersialylation or overexpression of sialyltransferases has been shown to either contribute to cancer cell metastasis or to be associated with cancer progression [18]. For example, sialyltransferase ST6Gal1, which adds the negatively charged sugar, sialic acid, to an α2,6 linkage at the terminal N-glycan, is overexpressed in many types of cancers such as colon cancer [19, 20]. Moreover, upregulation of ST6Gal1 is correlated with increased metastatic potential and poor prognosis in colon cancer as well as in other solid tumors [19, 21]. PDAC cells express high level of sialyl Lewis^x^ and possess high metastatic potential. In addition, overexpression of sialyltransferase ST3Gal3 can increase the level of sialyl Lewis^x^, thereby enhancing metastatic potential [22]. A recent proteomics study showed that metabolic flux can promote pancreatic cancer cells to become more aggressively malignant, possibly through increased protein sialylation [23]. Although hypersialylation contributes to cancer cell progression and metastasis, whether alterations in sugar supply can contribute to aberrant sialylation during cancer progression remains unknown.

This study was designed to determine the exact role of fructose in promoting pancreatic tumorigenesis. Additionally, we determined whether fructose can promote metastatic dissemination of PDAC. In order to determine if fructose intake directly contributes to tumor formation or passively promotes tumorigenesis, we crossed Kras^+/LSLG12D^ mice with Elas-CreER transgenic mice. The effect of fructose substitution was further evaluated utilizing human PDAC cell cultures. Orthotopic tumor-xenograft experiments were performed to determine whether fructose substitution can enhance the metastatic potential of PDAC cells. To explore the mechanisms underlying fructose-mediated enhancement of metastatic dissemination, RNA sequencing (RNAseq) analysis was conducted. In addition, the expression of ST6Gal1 and GLUT5 and their correlation with survival were investigated in patients with PDAC.

## RESULTS

### High-fructose diet promotes the development of aggressive pancreatic cancer

**Figure 1:**
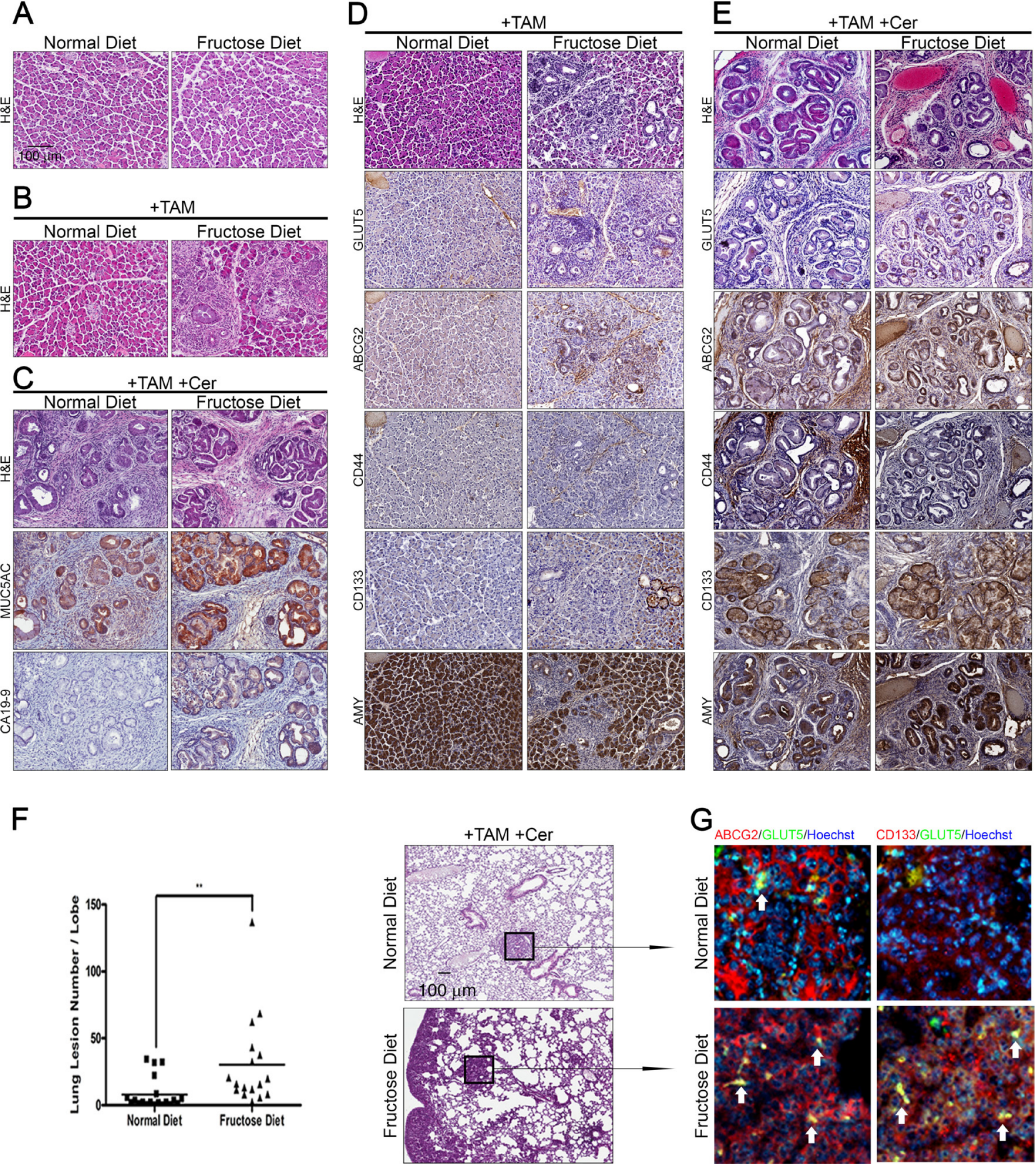
High fructose promotes the progression of advanced pancreatic cancer in Elas-CreER;Kras+/LSLG12D mice (**A** and **B**) Hematoxylin and eosin (H&E) staining shows histological changes in the pancreas under the indicated treatment in Elas-CreER;Kras^+/LSLG12D^ mice fed a normal or fructose diet. Tamoxifen (+TAM) was used to induce Kras^G12D^ activation (B). (**C**) Representative histological analysis of H&E, MUC5AC, and CA19-9 staining on pancreas sections of cerulein-treated Elas-CreER;Kras^+/LSLG12D^ mice (+TAM +Cer) fed a normal or fructose diet. (D and E) Serial paraffin sections of the pancreas from Elas-CreER;Kras^+/LSLG12D^ mice with Kras^G12D^ activation alone (**D**) or combined with cerulein treatment (**E**) were stained with antibodies against GLUT5, ABCG2, CD44, CD133, or amylase (AMY) antigen. (**F** and **G**) H&E staining of lung tissues, showing lung lesions in PanIN mice containing induced oncogenic Kras and combined with cerulein treatment. Lung lesions were counted and shown as the number of lesions per lobe (F). Immunofluorescence images of pulmonary tissues showing the distribution pattern of ABCG2 (red), CD133 (red), and GLUT5 (green) in lung lesions (G). The boxed area is magnified to better visualize the distribution and morphology of each staining pattern in lung lesions. **P < 0.01.

Although activated *Kras* mutation is the first genetic changes detected in about 40% of pancreatic intraepithelial neoplasia (PanIN) and in nearly 100% of PDACs [2, 24]. However, mutant Kras alone is insufficient to induce pancreatic tumorigenesis in adult mice. Previous work demonstrated that expression of *Kras*^G12V^ in adult acinar cells was not sufficient to induce neoplastic development unless mice underwent experimental pancreatitis induction [25]. We initially crossed Kras^+/LSLG12D^ mice with Elas-CreER transgenic mice, and confirmed that expression of *Kras^G12D^* mutant in adult acinar cells was not sufficient to induce pancreatic tumorigenesis (Figure 1A–1B) unless mice were treated with cerulein, an experimental pancreatitis inducer (Figure 1C). To determine whether fructose can promote pancreatic tumorigenesis, we fed Elas-CreER;Kras^+/LSLG12D^ mice treated with tamoxifen (+TAM) and with or without cerulein (+Cer) either standard laboratory chow (normal diet) or 60% fructose-enriched rodent chow (high-fructose diet) for 6 weeks (Figure 1B–1C, Supplementary Figure S1A). As shown in Figure 1A, in the absence of tamoxifen treatment (i.e., without induction of *Kras^G12D^* expression), no PanIN lesions was observed in control Elas-CreER;Kras^+/LSLG12D^ mice fed either a fructose diet or normal diet even after 10 weeks (Figure 1A, Supplementary Figure S1B). To induce *Kras^G12D^* expression in adult acinar cells, we pretreated 5-week-old Elas-CreER;Kras^+/LSLG12D^ mice with tamoxifen (+TAM). As expected, expression of *Kras^G12D^* in adult mice fed on a normal diet for 6 weeks did not induce the formation of PanIN (Figure 1B). In contrast, low-grade PanIN lesions were observed in the pancreas of *Kras^G12D^* -expressing mice fed a fructose diet for 6 weeks (Figure 1B).

As shown in Figure 1C, cerulein treatment of mice with the *Kras^G12D^* mutation induced PanIN and cancerous lesions (Figure 1C, +TAM+Cer). Compared to tamoxifen/cerulein-treated Elas-CreER;Kras^+/LSLG12D^ mice fed a normal diet, tamoxifen/cerulein-treated Elas-CreER;Kras^+/LSLG12D^ mice fed a high fructose diet for 6 weeks exhibited more high-grade PanIN lesions (as judged by the expression of Mucin 5AC) and adenocarcinomas (as judged by the expression of CA19-9 tumor antigen) (Figure 1C). To comprehensively characterize neoplastic lesions generated in tamoxifen/cerulein-treated Elas-CreER;Kras^+/LSLG12D^ mice fed either a normal diet or high fructose diet, we conducted immunohistochemical staining to examine the expression of acinar-cell markers-amylase (AMY) and GLUT5, as well as markers expressed in drug-resistant PDAC cells and/or pancreatic cancer stem cells, including ABCG2, CD44, and CD133 [26–28] (Figure 1D, 1E). Compared to tamoxifen or tamoxifen/cerulein-treated Elas-CreER;Kras^+/LSLG12D^ mice fed a normal diet, tamoxifen/cerulein-treated Elas-CreER;Kras^+/LSLG12D^ mice fed a high fructose diet presented with neoplastic lesions in the pancreas and expressed higher levels of GLUT5, ABCG2, and CD133 accompanied by aberrant expression of amylase. Moreover, extensive lung metastasis was observed in tamoxifen/cerulein-treated Elas-CreER;Kras^+/LSLG12D^ mice fed a high fructose diet (Figure 1F). Immunofluorescence staining further showed that abundant ABCG2-positive or CD133-positive subpopulation cells in lung nodules expressed GLUT5 (Figure 1G), indicating that invasive pancreatic cancer subpopulations are able to efficiently take up fructose.

### Fructose substitution in culture selectively enriches invasive ABCG2-positive subpopulations

Since invasive pancreatic cancer subpopulations in mice possibly possess the capability to take up fructose, we next evaluated whether fructose substitution has beneficial roles for invasive subpopulation of human PDAC cells. Four human PDAC cell lines, PANC-1, HPAC, PK1, and Pa8, were cultured in glucose-containing medium, and then transferred to medium containing 1g/L of fructose. Fluorescence-activated cell sorting (FACS) was performed to examine the expression of ABCG2, CD24, and CD44. Most PANC-1 cells expressed CD44, and contained four subpopulations: ABCG2+CD24+CD44+, ABCG2+CD24−CD44+, ABCG2-CD24+CD44+, and ABCG2-CD24−CD44+ (Supplementary Figure S2A). Of these four subpopulations, subpopulations expressing ABCG2 (ABCG2+CD24+CD44+ and ABCG2+CD24−CD44+) displayed drug resistance to gemcitabine and cisplatin and were highly invasive (Supplementary Figure 2B, 2C). To further examine the tumorigenicity and metastatic potential of each subpopulation, we subcutaneously or orthotopically engrafted 1 × 10^4^ cells of each subpopulation into NOD/SCID mice (Supplementary Table S1, Supplementary Figure S2D–S2E). Tumor xenoengrafts were allowed to grow for 3–4 months. Compared to unsorted cells, the ABCG2+CD24+CD44+ subpopulation exhibited greater tumorigenicity (Supplementary Table S1). In addition, ABCG2-positive subpopulations possessed higher metastatic potency as compared to ABCG2-negative subpopulations (Supplementary Figure S2D–S2E).

**Figure 2:**
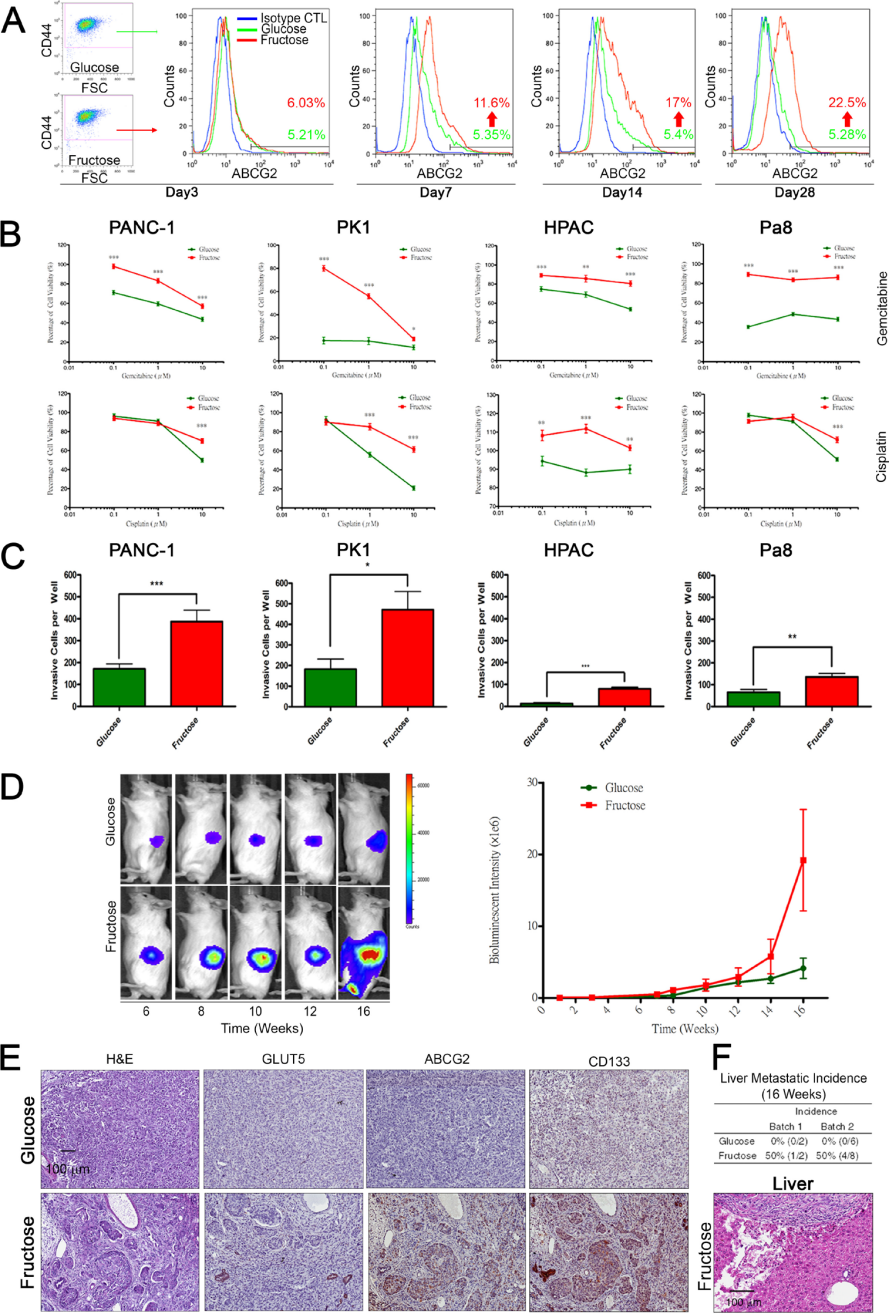
Long term fructose substitution gives rise to higher drug resistance and metastatic potential (**A**) Synchronization of PANC-1 cells grown in the indicated medium at different time points was analyzed by flow cytometry analysis based on ABCG2 and CD44 staining. Blue spots indicate cells stained with isotype control antibody as a negative control. (**B**) Representative cell viability results for pancreatic cancer cells, PANC-1, PK1, HPAC, and Pa8 cells, treated with fructose-substituted medium for 14 days, as measured by MTT assay with different concentrations of gemcitabine or cisplatin for 72 hours. (**C**) Pancreatic cancer cells treated with fructose-substituted medium for 14 days were examined by transwell assay, and representative bar figure show the invasive cells per well compared with parental cells. (**D**) *In vivo* imaging of PANC-1 cells in the NOD-SCID mouse showed gradual expansion of abdominal bioluminescent signals throughout the experimental period (left panel). The pooled results of the average bioluminescent signals are also shown in a graph (right panel). (**E**) Serial paraffin sections obtained from PANC-1-derived tumors were stained with H&E or with antibodies against GLUT5, ABCG2, and CD133. (**F**) Table summarizing liver lesion incidence in the two groups. Liver sections obtained from mice were analyzed by H&E staining. Values are shown as the mean ± SEM, *P < 0.05, **P < 0.01, ***P < 0.001.

A previous study indicated that the average level of fasting serum fructose was 5.7 ± 2.5 mM in patients with pancreatic cancer, which was higher than in healthy volunteers (1.9 ± 0.4 mM) [29]. To evaluate whether fructose substitution is beneficial for the growth of subpopulations exhibiting drug resistance and high invasion capability, we transferred PANC-1 cells to medium containing 1g/L of fructose (5.5 mM fructose) and allowed them to grow for 3, 7, 14, or 28 days (Figure 2A). Flow cytometric analysis revealed that fructose substitution culture increased the percentage of ABCG2-expressing PANC-1 cells from 5.2% to 22.5% (Figure 2A). Since ABCG2-expressing subpopulations displayed drug resistance and possessed high invasion capability, we examined whether a 14-day fructose substitution would enhance drug resistance and invasion capability. Four PDAC cell lines (PANC-1, HPAC, PK1, and Pa8) harboring either the *Kras* or *Nras* mutation (Supplementary Table S2) were transferred to medium containing 1g/L of fructose for 14 days. As shown in Figure 2B–2C, fructose substitution enhanced drug resistance and invasion capability of all four PDAC cell lines. To further determine if fructose substitution can affect the metastatic potential of PANC-1 cells, we orthotopically engrafted 1 ×10^4^ PANC-1 cells grown and passaged in either glucose- or fructose-containing medium for 4 weeks to NOD-SCID mice. All tumor-bearing mice were fed standard laboratory chow (normal diet). Tumor xenoengrafts were allowed to grow for 16 weeks, and *tumor progression* was monitored utilizing the Xenogen *IVIS* system. Quantification of bioluminescence intensity revealed that fructose substitution significantly enhanced the tumorigenicity of PANC-1 cells (Figure 2D). More ductular lesions formed in the tumor xenografts generated by PANC-1 cells grown under fructose-substituted conditions (Figure 2E, Supplementary Figure S3A). To further characterize neoplastic lesions generated by PANC-1 cells, we performed immunohistochemical staining to examine the expression of fructose transporter GLUT5, ABCG2, and CD133. Compared to tumor-engraftment generated by PANC-1 cells grown in glucose-containing medium, ductular lesions in the tumor engraftment generated by PANC-1 cells grown under fructose-substituted conditions expressed higher levels of GLUT5, ABCG2, and CD133 (Figure 2E and Supplementary Figure S3B–S3D). Moreover, histological analysis demonstrated that mice implanted with PANC-1 cells grown under fructose-substituted conditions presented several liver metastatic nodules (Figure 2F) indicating that fructose substitution selectively enriches invasive subpopulations of human PDAC cells.

### Fructose substitution upregulates ST6Gal1-mediated α2,6-sialylation

**Figure 3:**
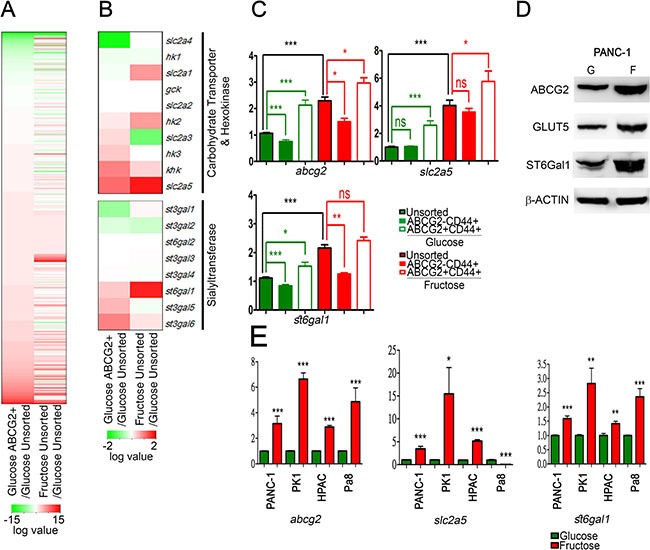
Fructose substitution increased ST6Gal1 (**A** and **B**) Hierarchical clustering analysis of gene expression patterns of PANC-1 cells grown under fructose substitution conditions for 28 days and the ABCG2-positive subpopulation were performed using R-project. Heat maps of whole gene (A) and carbohydrate transporter, hexokinase, and sialyltransferase gene sets (B) are shown as the log2 value of fold change compared with parental PANC-1 cells. (**C**) Relative mRNA expression of *abcg2*, *slc2a5* (GLUT5), and *st6gal1* were compared with those of unsorted PANC-1 cells. (**D**) Protein levels of ABCG2, GLUT5, and ST6Gal1 in PANC-1 cells cultured in glucose- (G) or fructose-substituted (F) medium; β-ACTIN was used as an internal control. (**E**) Expression levels of *abcg2*, *slc2a5*, and *st6gal1* in the indicated cells. Values are shown as the mean ± SEM, *P < 0.05, **P < 0.01, ***P < 0.001.

To identify the mechanisms underlying the promoting effect of fructose substitution on cancer metastasis, we performed RNA-seq analysis to analyze ABCG2-positive subpopulations and unsorted parent PANC-1 cells cultured under glucose-containing or fructose-substituted conditions for 4 weeks (Figure 3A). In general, 63653 gene reads were identified (Figure 3A) and transcriptome analysis was performed using the KEGG pathway dataset mapping revealed that higher levels of *slc2a5* (encoding GLUT5 transporter), ketohexokinases (*khk*), and hexokinase 2 (*hk2*) were present in ABCG2-positive subpopulations and unsorted parent PANC-1 cells cultured under fructose-substituted conditions (Figure 3B and Supplementary Table S3). The results imply that invasive ABCG2-positive subpopulations can uptake and metabolize fructose, perhaps accounting for how fructose substitution selectively enriches cells with drug resistance and high invasion capability. Transcriptome analysis further revealed higher levels of sialyltransferase *st6gal1* and *st3gal6* in both ABCG2-positive subpopulations and unsorted parent PANC-1 cells cultured under fructose-substituted conditions (Figure 3B). Real-time quantitative RT-PCR confirmed that ABCG2-positive subpopulations and unsorted parent PANC-1 cells cultured under fructose-substituted conditions for 2 weeks expressed higher levels of *abcg2*, *slc2a5*, and *st6gal1* (Figure 3C). Time-course experiments further showed that fructose substitution significantly increased the expression levels of *abcg2*, *slc2a5*, and *st6gal1* in a time-dependent manner (Supplementary Figure S4A–S4C). Western blot analysis confirmed that PANC-1 cells cultured under fructose-substituted conditions for 2 weeks expressed higher levels of ABCG2, GLUT5, and ST6Gal1 (marked as F in Figure 3D) compared to cells that were cultured in glucose-containing medium (marked as G in Figure 3D). The increase in the levels of *abcg2*, *slc2a5*, and *st6gal1* was also observed in PANC-1, PK1, and HPAC cells that harbored *Kras* and *p53* mutations (Figure 3E and Supplementary Table S2).

**Figure 4:**
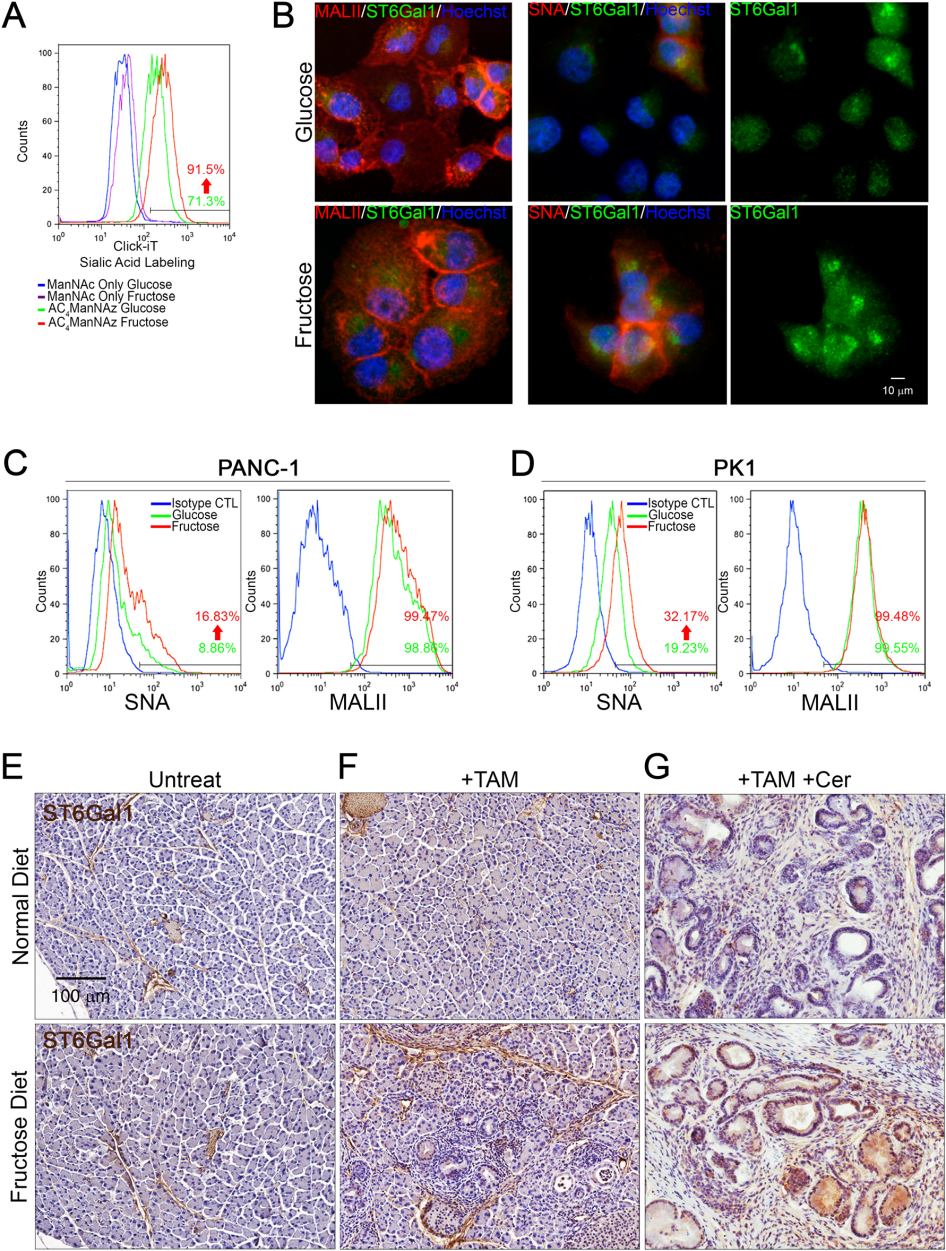
Fructose substitution increased α2,6-sialylation (**A**) Sialylated glycan profiles of parental (green) or PANC-1 cells cultured with fructose for 28 days (red) were detected by click-iT metabolic reagent labeling assays. Parental (blue) or PANC-1 cells cultured with fructose for 28 days (purple) were incubated with 400 nM ManNAc for 24 hours and then labeled with Alexa Fluor488 conjugated alkyne as a negative control. (**B**) Representative immunostaining results for ST6Gal1 (green), α2,6-sialylation (red), or α2,3-sialylation (red), as detected by using anti-ST6Gal1 antibody, biotinylated SNA, or MALII lectin, respectively. (**C** and **D**) Representative flow cytometry analysis showing staining against SNA and MALII in parental and PANC-1 (C) or PK1 cells (D) incubated for 28 days with fructose. Blue lines indicate cells stained with streptavidin-conjugated secondary antibody as negative control. (**E**–**G**) ST6Gal1 immunoreactivity and hematoxylin counterstaining on pancreas sections from control Elas-CreER;Kras^+/LSLG12D^ mice (E), and mice treated with tamoxifen (F) or tamoxifen/cerulein (G). Mice were fed a normal diet (upper panel) or fructose diet (lower panel).

Overexpression of sialyltransferases is associated with cancer progression and poor patient survival [19, 21]. Previous work demonstrated that overexpression of sialyltransferase, *st3gal3*, enhances the metastatic potential of PDAC cells [22]. Almaraz et al. further showed that the metabolic flux can cause pancreatic cancer cells to become more aggressively malignant via an increase in protein sialylation [23]. We therefore hypothesized that fructose substitution possibly leads to changes in cellular sialylation levels, which enhance pancreatic cancer cell mobility. To address this hypothesis, we used the click-iT metabolic labeling strategies [30] to investigate whether fructose substitution can increase sialylation. PANC-1 cells cultured in glucose-containing medium or fructose-substituted medium were pre-incubated with either N-Acetyl-D-mannosamine (ManNAc) or tetraacylated N-Azidoacetylmannosamine (ManNAc analog, Ac4ManNAz), and sialylation levels were detected by click azide/alkyne reaction. Flow cytometric analysis revealed that PANC-1 cells that had been cultured in fructose-substituted medium displayed higher fluorescence intensity compared to the control groups (ManNAc-treated cells or PANC-1 cells cultured in glucose-containing medium) (Figure 4A). These results indicate that PANC-1 cells cultured in fructose-substituted medium possessed higher sialylation activity.

To further determine whether fructose substitution can increase either α2,3-sialylation or α2,6-sialylation, we performed immunofluorescence staining using biotinylated Maackia Amurensis Lectin II (MALII) or biotinylated Sambucus Nigra Lectin (SNA) and anti-ST6Gal1 antibodies. As shown in Figure 4B, PANC-1 cells displayed intense MALII staining, and fructose substitution did not significantly enhance MALII staining. PANC-1 cells cultured under fructose-substituted conditions expressed higher levels of ST6Gal1 and displayed stronger SNA staining compared to cells grown in glucose-containing medium (Figure 4B). Flow cytometric analysis further validated that both PANC-1 and PK1 cells displayed strong MALII fluorescence, and that fructose substitution did not significantly enhance MALII fluorescence in either PANC-1 or PK1 cells (Figure 4C–4D). In contrast, PANC-1 and PK1 cells cultured under fructose-substituted conditions expressed higher levels of ST6Gal1 and displayed stronger SNA fluorescence compared to cells grown in glucose-containing medium (Figure 4C–4D). These results revealed that fructose substitution leads to an increase in α2,6-sialylation, possibly due to elevated levels of ST6Gal1. Compared to tamoxifen or tamoxifen/cerulein-treated Elas-CreER;Kras^+/LSLG12D^ mice fed a normal diet, mice fed a high fructose diet presented neoplastic lesions in the pancreas and these lesions expressed higher levels of ST6Gal1 (Figure 4E–4G). These findings confirmed the hypothesis that fructose upregulates ST6Gal1-mediated α2,6-sialylation, which possibly enhances pancreatic cancer cell mobility.

**Figure 5:**
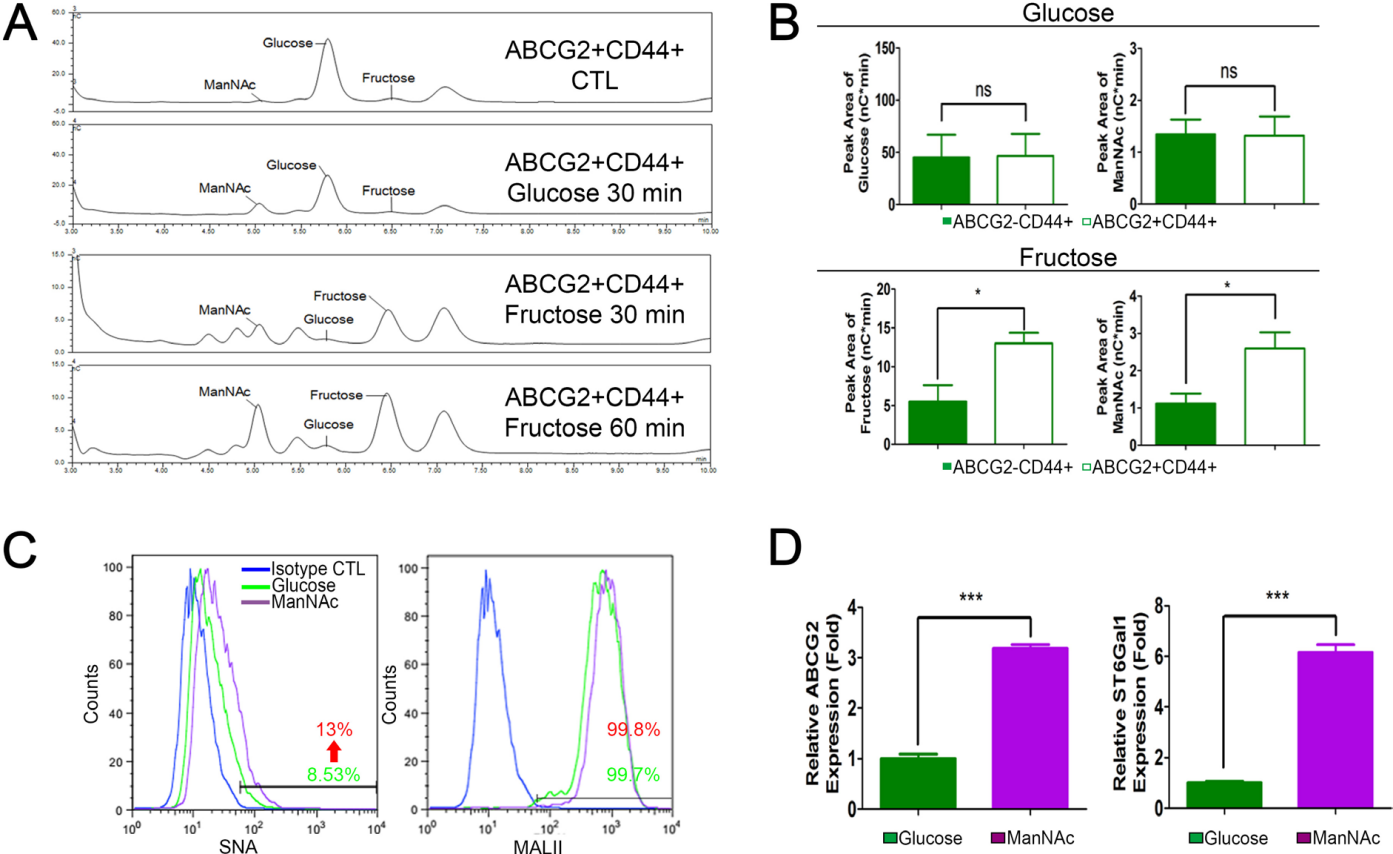
Fructose and ManNAc uptake contributes to high α2,6-sialylation (**A**) HPAEC spectra data show monosaccharide composition analysis of monosaccharide content release from cell extract of ABCG2+CD44+ subpopulation of PANC-1 cells cultured in the indicated conditions. ABCG2+CD44+ CTL indicates freshly isolated ABCG2+CD44+ subpopulation without any treatments. (**B**) Representative bar graphs of HPAEC show the peak area results of glucose and ManNAc from the indicated subpopulation incubated in glucose medium for 30 min (upper panel), or fructose and ManNAc peak area from the indicated subpopulation incubated in fructose medium for 60 min (lower panel). Data are from three independent experiments. (**C**) Representative flow cytometry analysis showing staining against SNA and MALII from PANC-1 cells grown in the indicated medium for 14 days. Blue lines indicate cells stained with streptavidin-conjugated secondary isotype control antibody as negative control. (**D**) Expression levels of *abcg2* and *st6gal1* in the indicated cells. Values are shown as the mean ± SEM, ns indicate non-significant, *P < 0.05, ***P < 0.001.

Previous work revealed exogenous ManNAc analogue can increase sialylation levels, leading to enhanced migration and invasion capability of pancreatic cancer cells [23]. High-performance anion exchange chromatography (HPAEC) was used to validate whether fructose uptake is associated with an increase in the generation of sialic acid precursor-ManNAc. As shown in Figure 5, higher levels of fructose and ManNAc can be detected in ABCG2-positive subpopulations cultured in fructose-substituted conditions (Figure 5A and 5B lower panel), confirming that ABCG2-positive cells could efficiently uptake fructose and produce ManNAc. Flow cytometric analysis further validated that ManNAc-substituted PANC-1 cells displayed strong SNA fluorescence compared to cells grown in glucose-containing medium (Figure 5C). We then determined if increased levels ManNAc caused ST6Gal1 upregulation. Real-time quantitative RT-PCR confirmed that PANC-1 cells cultured in ManNAc-substituted conditions for 2 weeks expressed higher levels of *abcg2* and *st6gal1* (Figure 5D). Thus, the results suggested that fructose utility enhanced sialic acid biosynthesis in ABCG2-positive subpopulations possibly through enhancing production of ManNAc. The increased level of ManNAc resulted in ST6Gal1 upregulation that led to increased α2,6-sialylation.

### ST6Gal1 is required for invasion and metastasis triggered by fructose substitution

**Figure 6:**
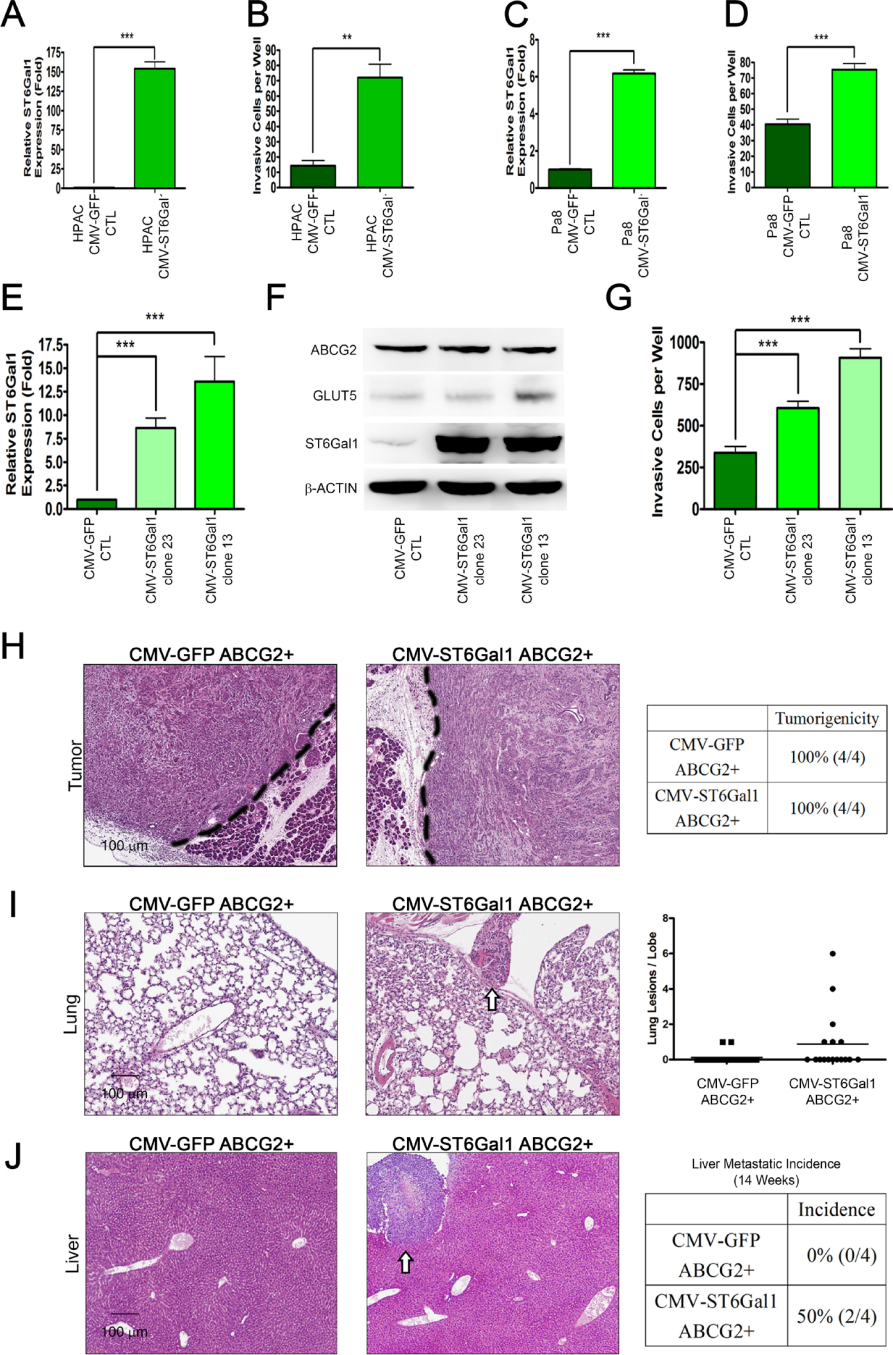
ST6Gal1 overexpression triggers pancreatic cancer metastasis (**A**–**D**) ST6Gal1 was transiently overexpressed in low invasive pancreatic cancer cells, Pa8 (A–B) and HPAC (C–D). The expression level of *st6gal1* was confirmed by real-time quantitative RT-PCR (A and C) and compared with that of cells expressing the control construct (CMV-GFP). The invasive ability conferred by both constructs was verified by matrigel-coated transwell invasion assay (B and D). (**E**) Real-time quantitative RT-PCR was performed to determine the relative mRNA expression of *st6gal1* in each clone stably overexpressing ST6Gal1 (clone 23 and 13) as compared with that in clones expressing the control construct (CMV-GFP CTL). (**F**) Protein levels of ABCG2, GLUT5, and ST6Gal1 (right panel) in each clone stably overexpressing ST6Gal1 (clone 23 and 13). (**G**) The invasive abilities of the control and stable clones overexpressing ST6Gal1 are shown as a bar graph. (**H**–**J**) H&E stain of orthotopic tumor (H), lung (I), and liver (J) sections from mice injected with the ABCG2-positive subpopulation of PANC-1 cells transformed with control vector (CMV-GFP ABCG2+) or ST6Gal1 construct (CMV-ST6Gal1 ABCG2+). Table summarizing the tumorigenicity in the two groups (H). White arrows indicate nodules in the lung (I) or liver (J). Lung lesions are shown as the number of lesions per lobe (I). Liver metastatic incidence is shown (J). Values are shown as the mean ± SEM, ns indicate non-significant, *P < 0.05, **P < 0.01, ***P < 0.001.

To determine whether ST6Gal1 upregulation by fructose substitution promotes pancreatic cancer metastasis, we investigated whether ST6Gal1 overexpression is sufficient to enhance the invasion capability of PDAC cells. Since HPAC and Pa8 cells displayed lower invasion capability compared to PANC-1 and PK1, we transiently overexpressed ST6Gal1 in HPAC (Figure 6A–6B) and Pa8 cells (Figure 6C–6D). As shown in Figure 6B and Figure 6D, ST6Gal1 overexpression was sufficient to enhance the invasive capability of HPAC and Pa8 cells. We also generated several stable clones of PANC-1 cells overexpressing ST6Gal1 at different levels. As shown in Figure 6E and Figure 6F, clone 13 and 23 expressed higher mRNA and protein levels of ST6Gal1 compared to parent PANC-1 control. To determine whether the invasion ability correlated with the level of ST6Gal1, we performed invasion assays with clone 13 and clone 23. Clone 13 and 23 expressed higher levels of ST6Gal1 as compared to the parent PANC-1 control displayed high invasion capability (Figure 6E–6G).

To further determine whether ST6Gal1 expression promotes the metastatic capability of the ABCG2-positive invasive population, we transiently overexpressed ST6Gal1, and isolated the ABCG2-positive subpopulation by FACS. We orthotopically injected 5 × 10^3^ ABCG2-positive cells (expressing either the CMV-GFP vector control or CMV-ST6Gal1 to overexpress ST6Gal1) into the pancreas of NOD/SCID mice. After 14 weeks, no significant difference in pancreatic tumorigenicity was observed through histological analysis (Figure 6H). Importantly, mice implanted with ST6Gal1-overexpressing ABCG2-positive cells presented lung and liver metastasis (Figure 6I–6J). Histological analysis demonstrated that mice engrafted with ST6Gal1-overexpressing ABCG2-positive cells presented several liver and lung metastatic nodules. In contrast, mice implanted with control ABCG2-positive cells did not form any lung or liver nodules. These results suggest that ST6Gal1 may play a key role in promoting lung and liver metastasis of ABCG2-positive PDAC cells.

**Figure 7:**
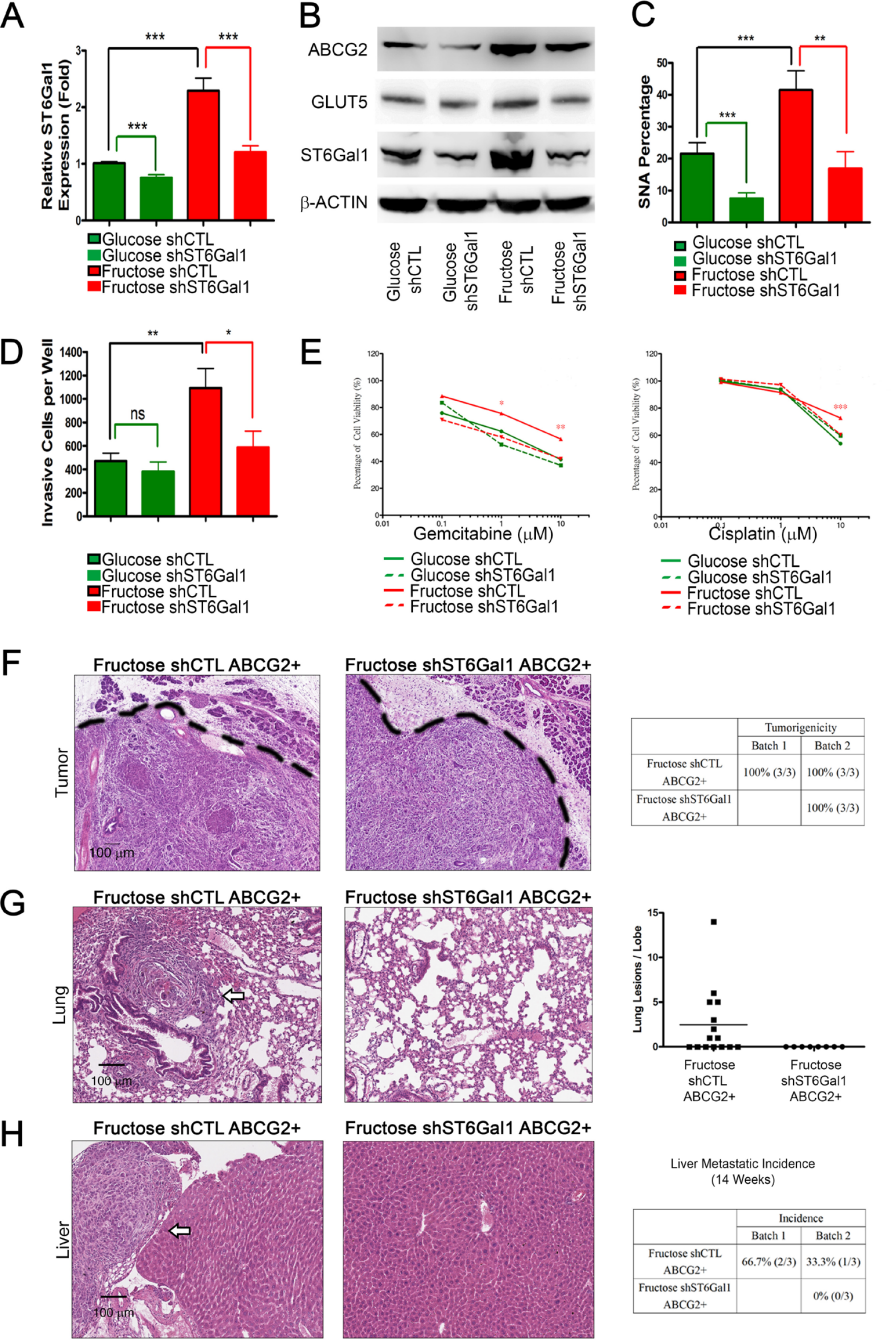
ST6Gal1 knockdown is sufficient to inhibit pancreatic cancer metastasis (**A**) Expression of *st6gal1* in the indicated cells. (**B**) Protein levels of ABCG2, GLUT5, and ST6Gal1 from indicated PANC-1 cell lysate were analyzed by western blotting; β-ACTIN was used as an internal control. (**C**) Flow cytometry analysis and SNA staining was performed to show α2,6-sialylation alteration under each condition. (**D**) Summary of the invasive ability of cells expressing each construct. Green lines: comparison with parental control (glucose shCTL); red lines: comparison with fructose control (fructose shCTL); black lines: comparison with parental control. (**E**) Cell viability of the indicated cells was measured by MTT assay with different concentrations of gemcitabine or cisplatin for 72 hours. (**F**–**H**) Histological analysis of orthotopic tumor (F), lung (G), and liver (H) tissues from mice injected with the indicated cells. Tables summarizing tumorigenicity (F) and liver metastatic incidence (H) of the indicated cells. Quantification of lung lesions detected by histological analysis (G). White arrows indicate metastatic lesions in lung (G) or liver (H) tissues. Values are shown as the mean ± SEM, ns indicate non-significant, *P < 0.05, **P < 0.01, ***P < 0.001.

To further determine whether ST6Gal1 mediates metastasis triggered by fructose substitution, we knocked down *st6gal1* using short hairpin RNA (shRNA). Real-time quantitative RT-PCR and western blotting analysis validated that fructose substitution increases mRNA and protein levels of ST6Gal1, and that shST6Gal can significantly decrease ST6Gal1 levels under both control and fructose-substituted conditions (Figure 7A–7B). The decrease in ST6Gal1 was accompanied by a reduction in levels of α2,6-sialylation (based on the reduction in the amount of PANC-1 cells that stained positively for SNA) (Figure 7C). The results of our transwell invasion and cell viability assay showed that *st6gal1* knockdown significantly suppressed the invasion potential and drug resistance of PANC-1 enhanced by fructose substitution (Figure 7D–7E). The suppression in the invasion potential and α2,6-sialylation elevated by fructose substitution was also observed in *st6gal1*-knockdown PK1 cells (Supplementary Figure S5).

Next, we isolated the ABCG2-positive subpopulation from PANC-1 cells cultured under fructose-substituted conditions for 4 weeks, and orthotopically injected 5 × 10^3^ ABCG2-positive cells (containing either shCTL (Fructose shCTL) or shST6Gal1 (Fructose shST6Gal1)) into the pancreas of NOD/SCID mice. After 14 weeks, histological analysis revealed no significant difference in pancreatic tumorigenicity (Figure 7F). Mice implanted with ABCG2-positive cells cultured under fructose-substituted conditions (Fructose shCTL) exhibited lung and liver metastases (Figure 7G–7H, Supplementary Table S4; compare with Figure 6I–6J). However, no lung or liver lesions was observed in mice implanted with shST6Gal-treated ABCG2-positive cells cultured under fructose-substituted conditions (Fructose shST6Gal1) (Figure 7G–7H). These findings imply that elevated ST6Gal1 mediates metastasis promoted by fructose substitution.

### Elevated ST6Gal1 expression is associated with poor prognosis in patients with PDAC

**Figure 8:**
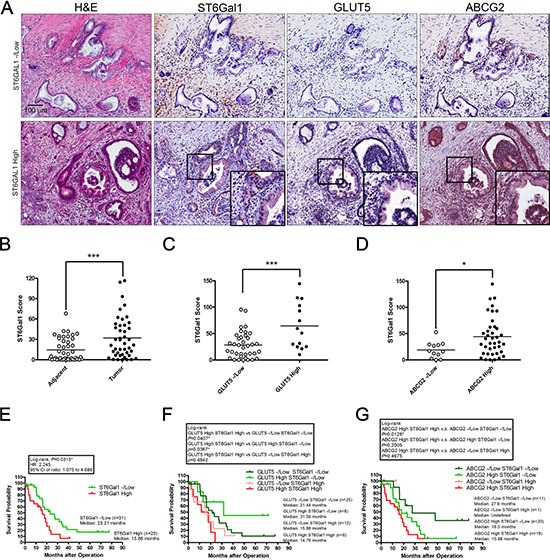
High ST6Gal1 expression correlates with poor outcomes in patients with pancreatic cancer (**A**) Serial sections were assessed by immunohistochemical staining with antibodies against ST6Gal1, GLUT5, and ABCG2. Representative images showed the histological features of samples with low or high ST6Gal1 expression. The expression level of ST6Gal1 was determined by scoring, and samples were classified into low and high expression groups. (**B**–**D**) The scores of ST6Gal1 staining were based on the percentage and intensity of positive immunohistochemical staining on epithelial-like tissue. Representative graphs showing the expression levels of ST6Gal1 in adjacent tissues and in tumor (B); correlations between ST6Gal1 and GLUT5 (C), or ST6Gal1 and ABCG2 (D). Horizontal bars represent the average values. (E-G) Kaplan–Meier survival analysis of the overall survival of patients with pancreatic cancer, stratified by the expression level of the following markers: ST6Gal1 (**E**), GLUT5 combined with ST6Gal1 (**F**), or ABCG2 combined with ST6Gal1 (**G**). *P < 0.05, **P < 0.01, ***P < 0.001.

To elucidate the clinical relevance of fructose intake in patients with PDAC, we collected 51 specimens from operable patients with PDAC who underwent pancreatoduodenectomy and analyzed expression of ST6Gal1, GLUT5, and ABCG2 in PDAC specimens through immunohistochemical (IHC) staining (Figure 8A). We compared 44 sets of matched samples from primary pancreatic tumors and their normal adjacent tissues. As shown in Figure 8B, tumor tissues expressed higher levels of ST6Gal1 than their normal adjacent tissues. No significant correlation was observed between ST6Gal1 levels and the patients’ age, serum CEA/CA19-9 levels, lymph-node metastases, or tumor–node–metastasis (TNM) stage (Supplementary Table S5). However, larger pancreatic tumors tended to express higher levels of ST6Gal1 (Supplementary Table S5). Importantly, Kaplan–Meier survival analysis showed that patients with PDAC expressing higher levels of ST6Gal1 exhibited significantly shorter survival (median survival: 15.88 months) as compared to patients with PDAC expressing lower levels of ST6Gal1 (median survival: 23.21 months) (Figure 8E).

We further performed IHC staining against ST6Ga1, GLUT5, and ABCG2, and divided samples into high and negative/low (-/low) expression groups based on the staining intensity and amount of positively stained areas in the epithelial structure of tumor lesions (Figure 8A, 8C, 8D). IHC staining conducted on serial sections in the high ST6Gal1 expression group revealed that GLUT5 and ABCG2 were expressed in the ST6Gal1-positive region (Figure 8A). Correlation analysis further indicated that high ST6Gal1 expression correlated with high levels of GLUT5 and ABCG2 expression (Figure 8C and 8D). Kaplan–Meier survival analysis further revealed that patients with PDAC expressing high levels of GLUT5 and ST6Gal1 exhibited significantly shorter survival (median survival: 14.76 months) than the other groups. Of the patients expressing high levels of GLUT5, those who also expressed low levels of ST6Gal1 exhibited the longest survival (median survival: 31.59 months) (Figure 8F). The results support our finding in animal studies that ST6Gal1 mediates the promoting effect of fructose on cancer metastasis (Figure 7F–7H). Patients expressing high level of both ABCG2 and ST6Gal1 exhibited shorter survival (median survival: 15.88 months) than patients expressing high levels of ABCG2 alone (Figure 8G). The results are consistent with our finding that invasive ABCG2-positive subpopulations expressing higher levels of ST6Gal1 were aggressive (Figure 6G–6I).

## DISCUSSION

PDAC is one of the most aggressive cancer types and is notorious for its tendency toward early metastasis [2]. Recent findings indicated that metastatic PDAC subpopulations possibly evolved from non-metastatic cell subsets at the tumorigenesis stage [31]. The current work revealed that dietary fructose promotes the development of aggressive pancreatic cancer in Elas-CreER;Kras^+/LSLG12D^ mice via upregulation of ST6Gal1-mediated α2,6-sialylation, thereby enhancing the metastatic potential of neoplastic cells. The findings raise the possibility that the metabolic flux may be a driving force to accelerate the evolution of non-metastatic cells into metastatic competent cells. Moreover, recent studies in which clonal subpopulations purified directly from patient biopsies were sequenced or metastatic nodules were analyzed through quantitative proteomic approaches demonstrated that clonal complexity of metastatic pancreatic cancer is already initiated within primary tumors [32, 33]. These findings can explain why PDAC undergo metastasis at an early stage. However, they did not explain the driving force behind early metastasis.

Indeed, cancer stem cells (CSCs) are proposed to persist in malignant tumors as a distinct population, and these cause relapse and metastasis by giving rise to new tumors. Evidence from recent work suggests that pancreatic cancer comprises several subsets of CSCs, including ESA+CD24+CD44+ cells, CD133+CXCR4+ cells, and c-Met+ subset cells, which are defined by their self-renewal ability, tumorigenicity, and metastatic ability [27, 28, 34, 35]. Furthermore, pancreatic cancer cells which express high levels of CD133, c-Met, or ABCG2 exhibit drug resistance [26, 28, 34, 35]. These studies imply that distinct subpopulations of cancer stem cells determine pancreatic cancer metastatic activity. The current work revealed that aggressive ABCG2-positive subpopulations exhibit selective outgrowth under fructose-substituted conditions. Tumors derived from PANC-1 cells cultured under fructose-substituted conditions expressed higher levels of fructose transporter GLUT5 and exhibited a significantly higher liver metastatic potential, even in an orthotopic xenograft mouse model fed normal rodent chow. Thus, our results support the hypothesis that high fructose intake possibly results in the reprogramming of the metabolic features of pancreatic cancer and increases cancer malignancy through selectively enriching subpopulations of human PDAC cells possessing high metastatic potentials.

A recent study also evaluated the effect of fructose on PDAC cells, reporting that pancreatic cancer cell lines exhibit similar cell proliferation rates after incubation in fructose-containing medium for 48 hours. The authors further showed that these PDAC cells entered the pentose phosphate pathway (PPP) after incubation, as revealed by increased expression of transketolase (including *tkt*, *tktl1*, and *tktl2*) [13], which are key enzymes of the non-oxidative pentose phosphate pathway responsible for production of ribose 5-phosphate for *de novo* nucleotide biosynthesis. Here, we found that ABCG2-positive cells passaged in either glucose- or in fructose-containing medium expressed high levels *tkt*, *tktl1*, and *tktl2* (Supplementary Figure S6A). In addition, 14-days of exposure to fructose-substitution medium enhanced *tktl1* expression in PK1, HPAC, and Pa8 cells (Supplementary Figure S6B). We also monitored the growth rate of pancreatic cancer cells passaged in glucose- or fructose-containing medium. Consistent with earlier findings [13], PANC-1 cells grew readily under both conditions (Supplementary Figure S6C). However, 14-day fructose substitution significantly prolonged the cell doubling time of PANC-1, PK1, HPAC, and Pa8 cells (Supplementary Figure S6C–S6D). The cell growth analysis showed that ABCG2-positive cells cultured under fructose-substituted conditions displayed a doubling time similar to that of cells cultured under glucose conditions, and ABCG2-negative cells presented a significantly prolonged doubling time (Supplementary Figure S6E). Furthermore, results of propidium iodide (PI) staining also illustrated that PI-positive populations in the fructose-substituted group were not significantly higher than those in cells cultured in glucose-containing medium. The findings exclude the possibility that fructose substitution selectively causes cell death (Supplementary Figure S6F). Thus, although fructose exposure resulted in enhanced expression of transketolase, culture for 14 days under fructose substitution conditions actually prolonged the cell doubling time of pancreatic cancer cells. The results suggest that transketolase upregulation may not be beneficial to PDAC cell growth.

Previous work showed that the growth rate of normal hamster ovary fibroblast CHO cells is limited by purine de novo biosynthesis, but not purine salvage biosynthesis [36]. Ong et al. examined metabolomic profiles by gas chromatography/mass spectrometry and liquid chromatography tandem mass spectrometry, and demonstrated that the purine de novo biosynthesis pathway can be substituted by the salvage pathway [37]. In contrast, nucleotide sugars are well-known activated forms of monosaccharide, and serve as a glycosyl donors in glycosylation. For example, CMP-N-acetylneuraminate (CMP-NeuNAc ) is an activated form of sialic acid, which catalyzes the transfer of a sialic acid residue to the terminus of an oligosaccharide chain of a glycolipid or glycoprotein [38]. Combined with the finding that fructose substitution upregulates α2,6-sialylation, these data suggest that fructose may serve as a biomaterial for the synthesis of sialic acid or nucleotide sugars, and not merely facilitate the synthesis of nucleotides for cell proliferation.

The link between the availability of different nutrients and glycosylation is a crucial mechanism linking metabolism and signaling. Enhancement of the carbohydrate modification response by nutrient utilization indicates that cells may adapt to different nutrient resources by alternate such modifications [39, 40]. Yarema *et al*. reported that addition of sialic acid in nutrient-deprivation or changing the metabolic flux by supplying an analog of ManNAc could increase sialylation levels and enhance the invasion capacity of cancer cells [23, 41]. Our quantitative HPAEC validation experiments showed that ABCG2-positive subpopulation could efficiently uptake and metabolize fructose to generate more ManNAc (Figure 5A–5B). Moreover, ManNAc-substituted PANC-1 cells displayed strong SNA fluorescence and expressed higher levels of *abcg2* and *st6gal1* compared to cells grown in glucose-containing medium (Figure 5C–5D). The findings suggested that fructose utility enhanced sialic acid biosynthesis in ABCG2-positive subpopulations possibly through enhancing production of ManNAc. The increased level of ManNAc resulted in ST6Gal1 upregulation that led to increased α2,6-sialylation.RNA-seq data also showed that the ABCG2-positive subpopulation originally expressed higher levels of ST6Gal1, and our overexpression and knockdown studies revealed that ST6Gal1 crucially regulates the invasion ability of pancreatic cancer cells, especially in the ABCG2-positive subpopulation. Thus, we suggest that ST6Gal1 mediates the promoting effect of fructose on pancreatic cancer metastasis.

In conclusion, the current study provides new insights explaining the association between high fructose intake and increased pancreatic cancer risk. We revealed that invasive ABCG2-positive subpopulations could be selectively outgrown under fructose-substituted conditions and elucidated a crucial role of ST6Gal1 in elevating the invasiveness of PDACs in a fructose-responsive manner. Since Martin et al. reported that there was no significant histological abnormality in the organs of ST6Gal1 null mice [42], we anticipate that sialyltransferase ST6Gal1 can serve as a potential target for future pancreatic cancer therapeutics development.

## MATERIALS AND METHODS

### Kras knock-in and orthotopic mouse model of pancreatic cancer

All animal experiments were approved by the Academia Sinica Institutional Animal Care and Utilization Committee. Elas-CreER transgenic mice were generated at the Level Transgenic Center (Taipei, Taiwan) as described in as our previous work [43]. Kras^+/LSLG12D^ mice (B6;129-Kras2 <tm4Tyj>) bearing a Cre-dependent conditional knock-in mutation Kras^G12D^ were imported from Mouse Models of Human Cancer Consortium [44]. Kras^+/LSLG12D^ mice were bred with Elas-CreER mice to generate Elas-CreER;Kras^+/LSLG12D^ mice. To induce Kras^+/LSLG12D^ in acinar cells, 5-week-old Elas-CreER;Kras^+/LSLG12D^ mice were intraperitoneally injected with free base tamoxifen (20 mg/mL in corn oil; Sigma-Aldrich) three times per week (3 injections of 2 mg each). Following induction of Kras^+/LSLG12D^ by tamoxifen injection for 1 week, cerulein mice were intraperitoneally injected with caerulein (50 μg/mL in PBS; Sigma-Aldrich) six times per week (6 injections of 5 μg each) to induce chronic inflammation. Than animals were provided with *ad libitum* access to a normal diet (Cat: 5010, Laboratory Animal Care Course) or a high fructose diet (60% fructose diet, Cat: TD96130, Harlan Laboratories). The various treatments for the Kras knock-in model used in this study are described in Supplementary Figure S1A.

Orthotopic mouse model of pancreatic cancer was generated based on the method Kim *et al* previously described [45]. Briefly, pancreatic cancer subpopulations were freshly isolated by FACSAriaII cell sorter (BD Biosciences). The left abdominal region of NOD/SCID mice was shaved using electric clippers and then abdominal skin was incised using sterile microscissors. And then the underlying muscle was grasped and lift with forceps and was incises to enter the abdominal cavity. Using a pair of blunt-nose forceps to gently grasp the tip of the pancreatic tail and externalize the pancreas/spleen in a lateral direction. FACS-sorted cells were mixed with 20 μl matrigel and then slowly injected into pancreas. The incision was closed with 0.1 mm (5/0) SAFIL synthetic absorbable structure silk stitches (B. Braun, Spain). All procedures were manipulated in a sterile ventilated hood. The pancreatic tumor growth was monitored using IVIS Spectrum *In Vivo* Imaging System (PerkinElmer).

### Flow cytometric analysis

Human pancreatic cancer cells were dissociated with trypsin, washed twice with HBSS containing 2% fetal bovine serum and 10 mM HEPES (staining buffer). Cells were stained and incubated on ice for 30 minutes with the following primary antibodies: ABCG2 (R&D Systems Inc, 1:50), FITC-conjugated CD24 (BD Biosciences, 1:100), APC-conjugated CD44 (BD Biosciences, 1:100), biotinylated SNA lectin (Vector Laboratories, 1:100), or MALII lectin (Vector Laboratories, 1:100). After stained with appropriated secondary antibody, the stained cells were analyzed by FACSCalibur (BD Biosciences).

### Histology and immunohistochemistry

Tissues were fixed overnight at room temperature with 3.5% formaldehyde solution containing 68.6% EtOH and 4.8% acetic acid (FAA fixation) prior to being processed and embedded in paraffin. Four micrometer thick sections were cut and mounted on Superfrost plus slides (Thermo Scientific). For immunohistochemical staining, sections were subjected to antigen retrieval in antigen unmasking solution (Vector Laboratories) for 30 minutes at 95°C in a water bath. The sections were then incubated in 2% blocking buffer (Roche). Primary antibodies were used at the following dilutions: CD44 (Santa Cruz, 1:100), ABCG2 (human specimen: R&D, 1:50; mouse tissue: Santa Cruz, 1:50), GLUT5 (Thermo Fisher Scientific, 1:50), CD133 (abcam, 1:100), ST6Gal1 (IBL, 1:100), GFP (Invitrogen, 1:100), and HRP-conjugated secondary antibodies. The HRP signal was amplified using the Super Sensitive™ IHC Detection System (BioGenex). Sections were counterstained with haematoxylin (Muto Pure Chemicals LTD., Tokyo, Japan), dehydrated, and mounted with Malinol (Muto Pure Chemicals LTD.). Slides were examined with ScanScope Digital Slide Scanners (Aperio, Vista, CA). Positive staining was scored using the following formula as previous described [46]:

Where t is the total area of epithelial-like tissue, r3 is the total area of high intensity staining (intensity 3), r2 is the total area of medium intensity staining (intensity 2), and r1 is the total area of weak intensity staining (intensity 1).

### Case selection

A total of 51 patients diagnosed with PDAC at Taipei Veterans General Hospital from 2008 to 2014 were included in this study. All patients received standard treatment protocols according to hospital guidelines. Patients with operable PDAC underwent pancreatoduodenectomy. Clinical information, pathology data, and follow-up data were collected via retrospective review of the medical records; the longest clinical follow-up time was 90 months. All cases were staged according to TNM classification of malignant tumors of the American Joint Committee on Cancer, and histological cancer types were identified based on World Health Organization classification. Overall survival was defined as the interval after treatment to death. The study was carried out with the approval of the Institutional Review Board and with permission from the ethics committees of the institutions involved (AS-IRB02-101149).

Pa8 cells were derived from a primary specimen from a 65-year-old female patient with operable PDAC provided by the Taipei Veterans General Hospital, and all experiments involved cells passaged 15–35 times. PK1 cells were derived from the pleural effusion of a 53-year-old male patient with metastatic PDAC, which was provided by National Taiwan University Hospital; all experiments were used cells passaged 20–40 times.

### Cell culture conditions

Human pancreatic cancer cell lines PANC-1 and HPAC were purchased from the Bioresource Collection and Research Center (Hsinchu, Taiwan). PANC-1 and Pa8 cells were cultured in DMEM containing 4.5 g/L glucose, 100 units /ml penicillin and streptomycin, and 10% fetal bovine serum. PK1 cells were cultured in DMEM containing 4.5 g/L glucose, 100 units /ml penicillin and streptomycin, 10% fetal bovine serum, 10 ng/ml EGF (PeproTech), 10 ng/ml bFGF (PeproTech), and ITS (Insulin-Transferrin-Selenium; Invitrogen). HPAC cells were cultured in DMEM F-12 (USBiological) containing 3.151 g/L glucose, penicillin and streptomycin, and 10% fetal bovine serum. To study the effect of fructose replacement, glucose in the basal medium was replaced with 1g/L of fructose (Wako, Osaka, Japan).

### RNA sequencing analysis

To assess RNA-seq of PANC-1 subpopulations, total RNA was extracted with Trizol reagent (Invitrogen) and the RQ1 DNase digestion kit (Promega), according to the manufacturer's instructions. Ten to fourteen million RNA-Seq reads were obtained, of which 200-700 nt fragmented mRNA samples were treated with an Illumina TruSeq RNA v2 kit (Non-Stranded) according to the manufacturer's protocol, and then analyzed by Welgene Biotech Co. (Taipei, Taiwan). Briefly, the reads were initially trimmed according to quality score and base ambiguity. Moreover, these processed reads were mapped to the human RefSeq genomic sequence with annotations. Then the mapped gene reads were represented as reads per kilobase of exon per million mapped reads (RPKM). R-project was used to display the heat map of each gene set.

### RT-PCR, and real-time quantitative RT-PCR (qPCR)

5 μg of total RNA was reverse transcribed into complementary DNA (cDNA) by oligo (dT) primer using a RevertAid RT Kit (Thermo Scientific) according to the manufacturer's protocol. Of the obtained cDNA, 20 ng were used as template in qPCR with the ABI PRISM 7900HT sequence detection system (Applied Biosystems). The relative expression levels were normalized to *gapdh*. The primers are described in Supplementary Table S6.

### Western blotting analysis

The cells were lysed with RIPA buffer (20 mM MOPS pH 7, 150 mM NaCl, 1 mM EDTA, 1% NP40,1% Deoxycholic acid, 0.1% SDS) for 30 min on ice. Samples were sonicated, separated by SDS-PAGE (10%), and subsequently transferred to PVDF membrane (Millipore). Samples were incubated in blocking buffer (0.1% Tween 20, 5% nonfat dry milk in TBS buffer) for 1 hour at room temperature. Afterwards, the membrane was incubated with primary antibody in blocking buffer overnight at 4°C. Then washed twice with TBST (0.1% Tween in TBS) and incubated with the appropriate secondary antibody in blocking buffer for 1 hour at room temperature. The blot was developed using the Immobilon^TM^ Western Chemiluminescent HRP substrate (Millipore). The primary antibodies were used anti-ABCG2 (OriGene Technologies, 1:1000), anti-GLUT5 (GeneTex Inc., 1:1000), anti-ST6Gal1 (Invitrogen 1:200), anti-β-ACTIN (Sigma-Aldrich, 1:2000).

### Sialylated glycan labeling

To label sialic acid with Click-iT^®^ metabolic reagent (Invitrogen), cells were pre-incubated with 400 nM Ac4ManNAz (Invitrogen) or negative control substrate 400 nM ManNAc (Sigma-Aldrich) for 24 hours and the cells were labeled with Alexa Fluor488 conjugated alkyne for detection. After incubation for 30 minutes at room temperature, cells were washed and analyzed using FACSCalibur (BD Biosciences). All labeling procedures were performed based on the manufacturer's protocol with modifications as previously described [30].

### Immunofluorescence staining

Cells fixed with 4% paraformaldehyde, and permeabilized at room temperature in 0.1% Triton X-100 for 30 minutes. After blocking with blocking buffer (Roche), the cells were incubated with primary antibody overnight at 4°C and with secondary antibody for 2 hours at room temperature. The primary antibodies were used at the following dilutions: anti-ST6Gal1 (ST6Gal1-M2, IBL, 1:50), SNA (Vector Laboratories, 1:100), MALII (Vector Laboratories, 1:100), and ABCG2 (R&D, 1:50). Cells were counter-stained with Hoechst dye (Sigma-Aldrich) to visualize the cell nuclei. Immunostaining images were obtained using a fluorescence microscope (Leica Microsystems Inc).

### Monosaccharide detection

HPAEC was used to detect the monosaccharides uptake ability of cancer cells by Dionex™ ICS-3000 DC (Thermo Scientific). Briefly, 2 × 10^5^ indicated subset of PANC-1 cells were freshly isolated by cell sorting, incubated in the indicated medium, washed in PBS, and sonicated in 300 μL of 50 mM ammonium bicarbonate, pH 8.0. Cell lysate was centrifuged at 13000 × *g* for 10 min and protein was precipitated by adjusting the supernatant to 70% ethanol. Supernatant samples were evaporated, resuspended in 150 μL H_2_O, and analyzed on a CarboPac PA1 (2 × 250 mm) column. Chromatographic elution was carried out at a flow rate of 0.25 mL min^−1^ using 100 mM NaOH for 0–10 min, and the application of a linear gradient after 10 min to 500 mM sodium acetate over 20 min. The separated monosaccharides were detected using pulsed amperometric detection (PAD) with a gold working electrode.

### Generation of ST6Gal1 overexpression and ST6Gal1 knockdown cells

The pCMV6-AC-GFP-ST6Gal1 vector (Origene) was transfected into the indicated cancer cells using polyJet transfection reagents (SignaGen) and incubated in G418 (MDBio)-containing medium. For transient expression of ST6Gal1, cells were assessed at fewer than 14 days after transfection. Clones stably expressing ST6Gal1 were generated by seeding cells into 6-well plates at a very low density. Clones derived from a single cell were picked up by tip and amplified.

Lentivirus carrying shRNA of ST6Gal1 was obtained from the RNAi core at the Genomics Research Center. The target sequences of shRNA are listed in Supplementary Table S7. Equal aliquots of lentivirus supernatants containing ST6Gal1 were added to 6-well plates and incubated for 24 hours. On day 3, these cells were cultured in puromycin (MDBio)-containing medium and all of the cells were assessed at fewer than 14 days after infection.

The levels of ST6Gal1 and α2,6-sialylation were clarified by qPCR and flow cytometry analysis.

### Statistical analysis

All *in vitro* observations were confirmed by at least three independent experiments. Data were analyzed using the Prism 5 software program (GraphPad Software, San Diego, CA). All values are presented the as mean ± standard error of the mean (SEM). Two-tailed, unpaired Student's *t* tests were used for pair-wise comparisons. Survival curves were analyzed by the log-rank test in Kaplan–Meier survival analysis. Chi-square test for categorical variables was used for comparing the tumorigenicity, metastatic incidence, or clinicopathological features between two groups. *P*-values of less than 0.05 were considered to be significant.

## SUPPLEMENTARY MATERIALS FIGURES AND TABLES




