# Supplementary material for: Elevation of β-galactoside α2,6-sialyltransferase 1 in a fructose-responsive manner promotes pancreatic cancer metastasis
Source: Oncotarget. 2016 Dec 9;8(5):7691–709. doi: 10.18632/oncotarget.13845 (PMC5352353; doi:10.18632/oncotarget.13845)
Supplement: Supplementary file 1 [file oncotarget-08-7691-s001.pdf]

Elevation of β-galactoside α2,6-sialyltransferase 1 in a fructose-responsive manner promotes pancreatic cancer metastasis

Supplementary Materials

DNA sequence analysis

DNA from PANC-1, PK, and HPAC cells was extracted using a Proteinase K-based DNA extraction protocol. Tri-I Biothech Inc. (Taipei, Taiwan) constructed a DNA library using an Illumina TruSeq Amplicon-Cancer Panel Library Prep Kit, and generated 2 × 150 bp paired-end sequences with MiSEQ. DNA sequences were trimmed by CLC-GWB v5.1, and low quality sequences, ambiguous nucleotides, and terminal nucleotides were removed.

Cell viability and drug resistance

Cells were seeded at 1×10^4 cells/well in 96-well plates. Different concentrations of gemcitabine (United States Pharmacopeia, Rockville, MD) and cisplatin (Calbiochem, Darmstadt, Germany) were added to the wells and incubated for 72 hours. Cells were washed once with PBS, and then incubated with 3-(4,5-dimethylthiazol-2-yl)-2,5-diphenyltertrazolium bromide (MTT, Sigma, 1 mg/mL, 50 µL per well) at 37°C for 2 hours, and then dimethylsulphoxide (150 µL per well) was added at room temperature. The color reaction was examined using an ELISA reader at 570 nm.

The survival ability of each subpopulation was determined using the following formula:

(n2 × n2%)/(n1 × n1%).

Where n2 is the cell number of each subpopulation after chemotherapeutic drug treatment for 72 hours, n2% is the percentage of each subpopulation in the population after chemotherapeutic drug treatment; n1 is the cell number of each subpopulation without treatment after 72 hours, and n2% is the percentage of each subpopulation in the population without treatment after 72 hours. Based on the IC_{50} results of PANC-1 (Figure 2B), we used 1 µM gemcitabine and 10 µM cisplatin to examine the survival ability of pancreatic cancer cells.

Transwell invasion assay

A sample of 1×10^4 of the indicated cancer cells was resuspended in 400 µL serum-free medium and placed into the top chamber of an insert (Millipore) pre-coated with 400 µg/ml Matrigel. The lower chamber of the well was filled with serum-containing culture medium. After 24 hours of incubation, cells that did not invade were removed by a cotton swab, and cells on the lower surface of the membrane were fixed with methanol for 20 minutes, and then stained with Giemsa's solution (Merck KGaA, Darmstadt, Germany). The number of invasive cells per well was counted under a light microscope.

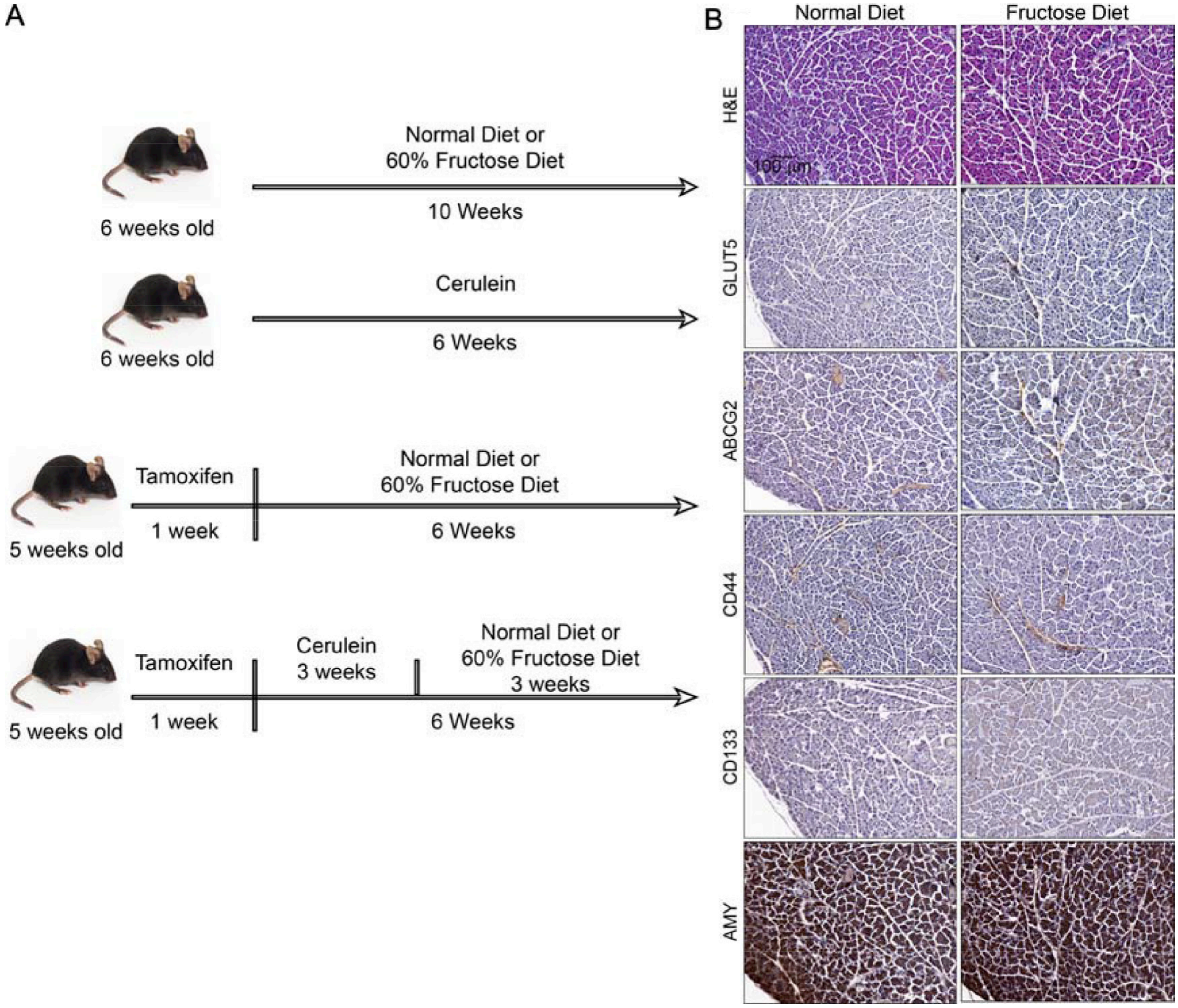

Supplementary Figure S1: Expression profiles of GLUT5, ABCG2, CD44, CD133, and amylase in control Elas-CreER;Kras$^{+/LSLG12D}$ mice. (**A**) Summary of the various treatments and time points used in the Elas-CreER;Kras$^{+/LSLG12D}$ mouse study. (**B**) Expression of GLUT5, ABCG2, CD44, CD133, and amylase (AMY) in pancreatic tissue sections from Elas-CreER;Kras$^{+/LSLG12D}$ mice.

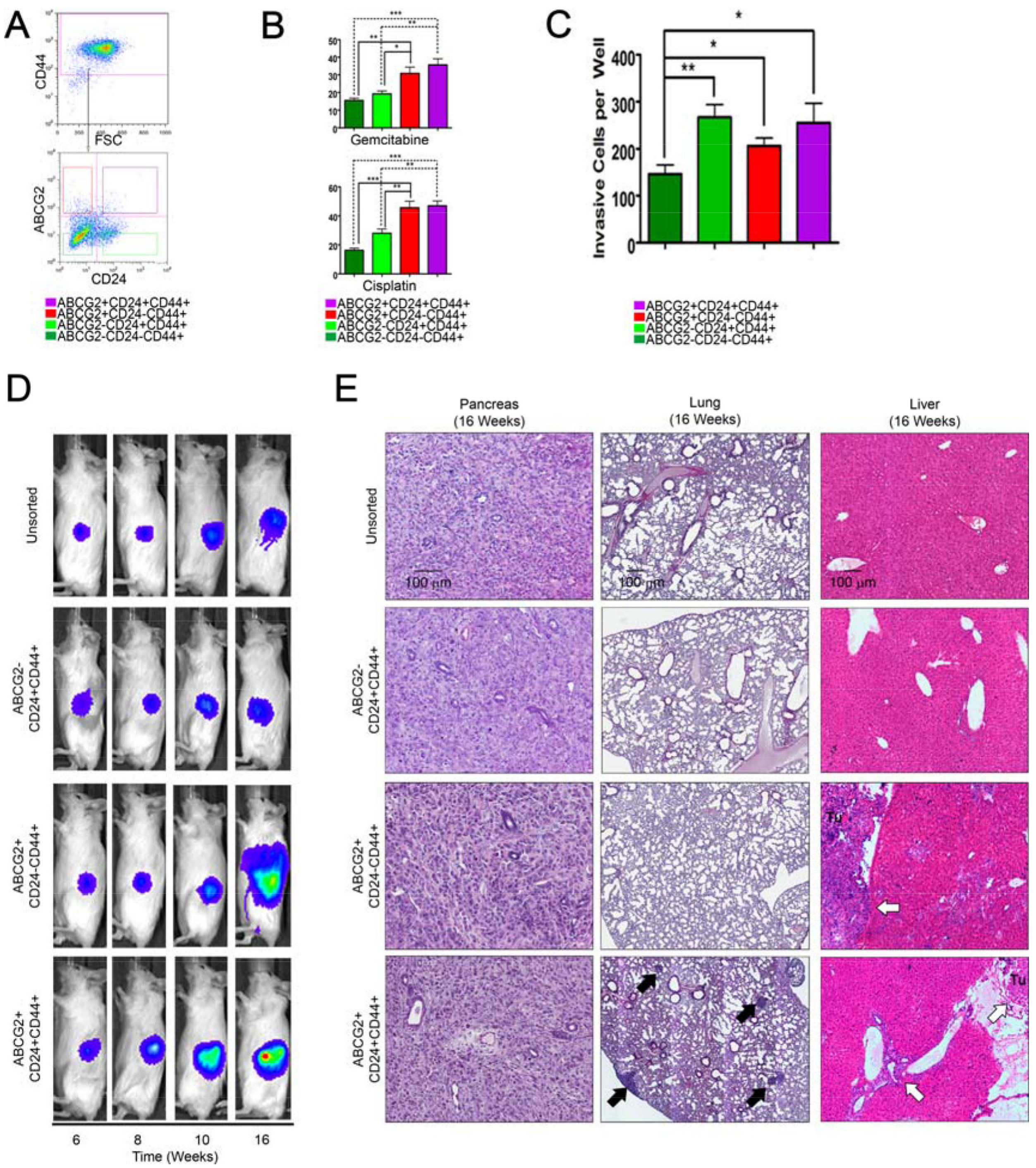

Supplementary Figure S2: The ABCG2-positive subpopulation possesses higher drug resistance and metastatic potency. (**A**) Flow chart depicting the method using to isolate each subpopulation of PANC-1 cells (cells were stained with antibodies against ABCG2, CD24, and CD44). (**B**) PANC-1 cells were treated with two standard chemotherapeutic drugs, gemcitabine and cisplatin, for 72 hours. ABCG2, CD24, and CD44 were analyzed by flow cytometry. Representative data show the fold change of cell number of each subpopulation after treatment versus ABCG2+CD24−CD44+ (solid line) or ABCG2+CD24+CD44+ (dashed line). (**C**) Freshly isolated subpopulation cells were assessed by transwell assay, and representative bar graphs show the invasive cells per well compared with the ABCG2−CD24−CD44+ subpopulation. (**D**) Representative bioluminescence IVIS images of animals in the four groups are shown from week 6 to week 16. (**E**) Pancreatic tumor, lung, and liver sections of unsorted PANC-1 cells, ABCG2−CD24+CD44+, ABCG2+CD24−CD44+, and ABCG2+CD24+CD44+ cells were histologically analyzed by H&E staining. Tu indicates tumor. Black arrows indicate lung lesions and white arrows indicate liver lesions. Values are shown as the mean ± SEM, *$P < 0.05$, **$P < 0.01$, ***$P < 0.001$.

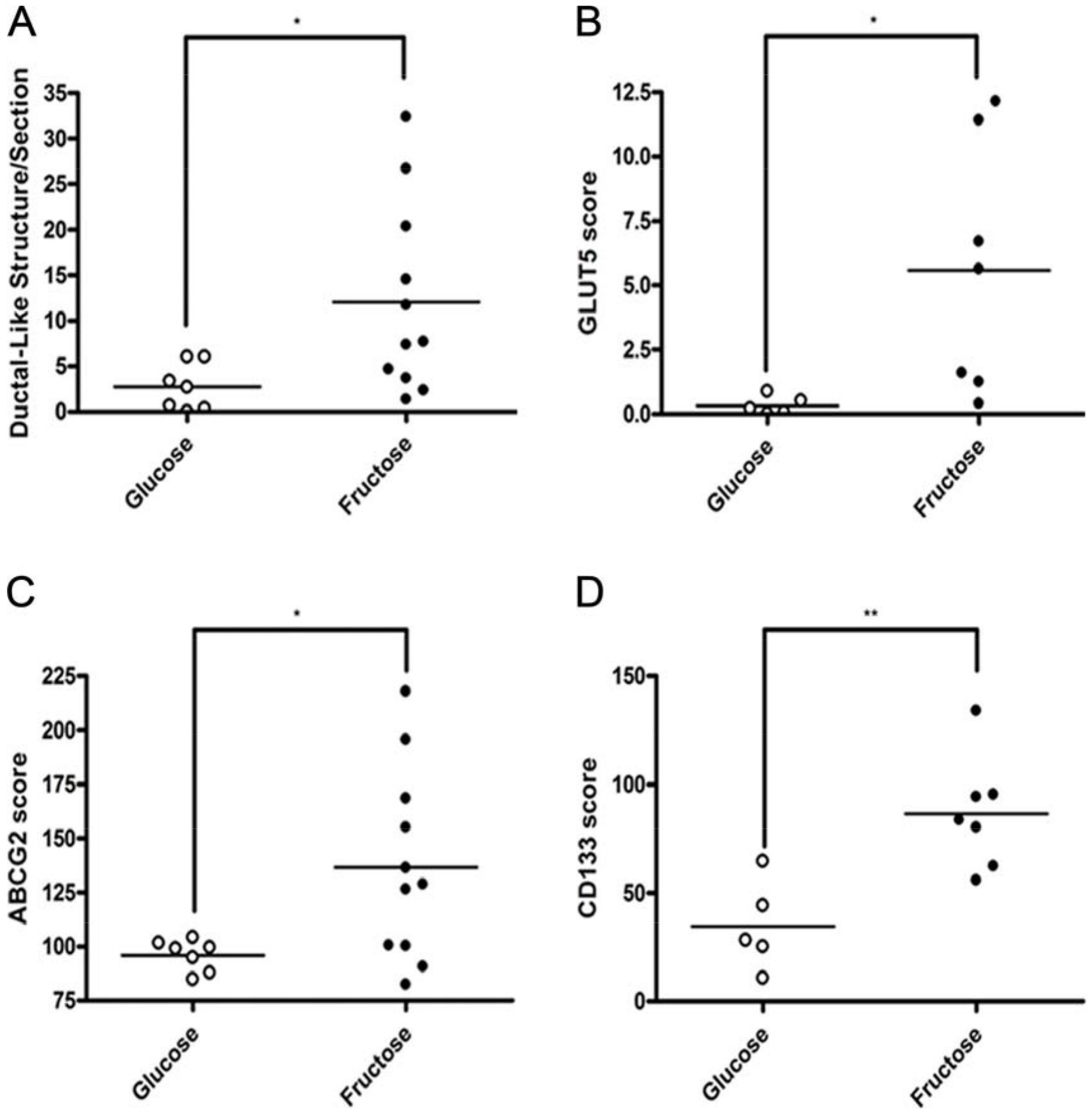

Supplementary Figure S3: Histological analysis of ductular lesions and immunohistochemistry scoring of GLUT5, ABCG2, and CD133. (**A–D**) Representative histological scoring graphs reveal ductular lesions (A), GLUT5 (B), ABCG2 (C), and CD133 (D) in parental (glucose) and 28-day-fructose substituted (fructose) PANC-1 cell-derived tumor tissue sections. Values are shown as the mean ± SEM, $^{*}P < 0.05$, $^{**}P < 0.001$.

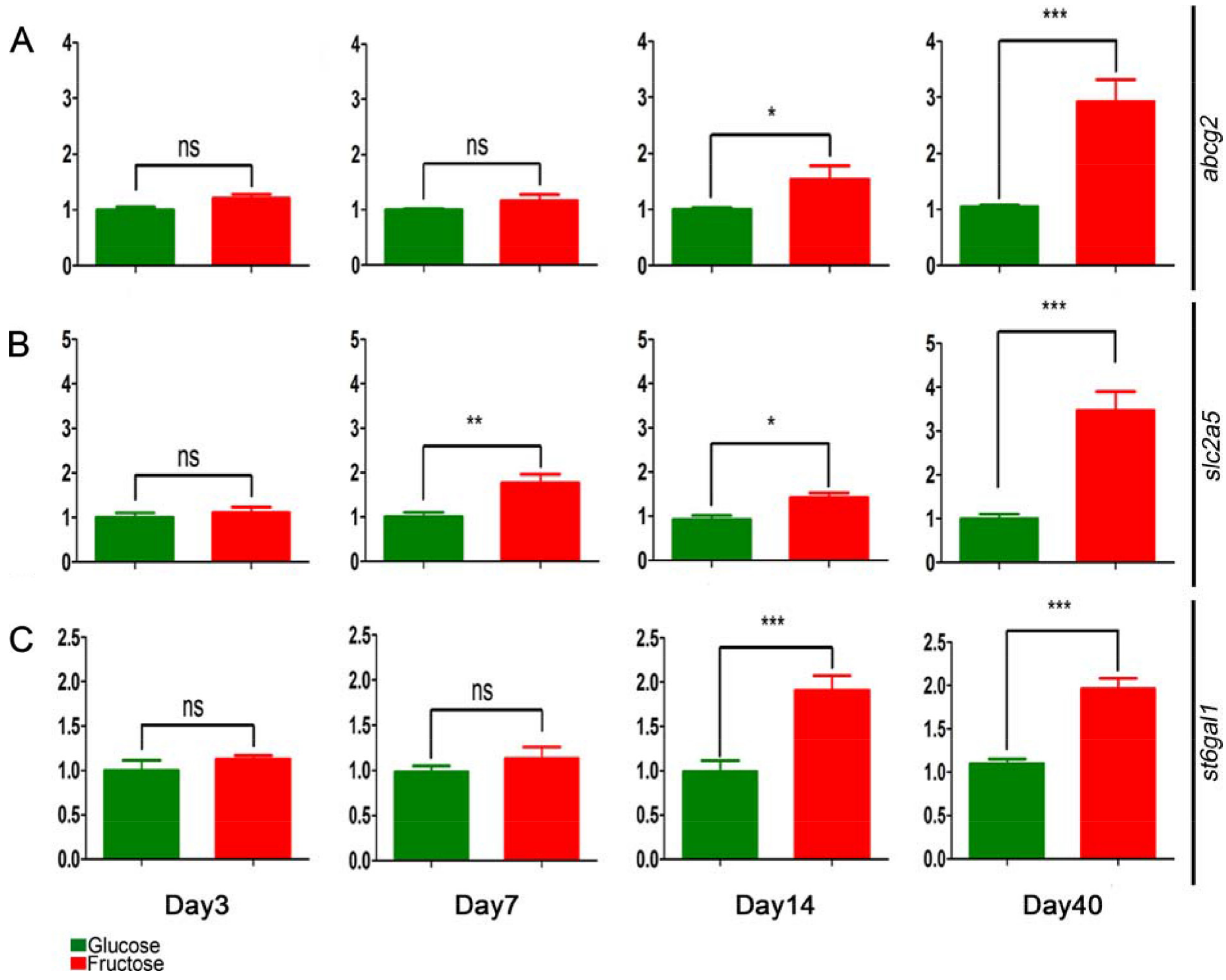

Supplementary Figure S4: Time-dependent increase of *abcg2*, *slc2a5*, and *st6gal1* levels in fructose-substituted PANC-1 cells. (**A**–**C**) Synchronized PANC-1 cells grown and passaged in glucose- (green) or in fructose-containing medium (red) were examined at the indicated time points for the expression of *abcg2* (A), *slc2a5* (B), and *st6gal1* (C) by real-time quantitative RT-PCR. Values are shown as the mean ± SEM, ns indicates non-significant, *$P < 0.05$, **$P < 0.01$, ***$P < 0.001$.

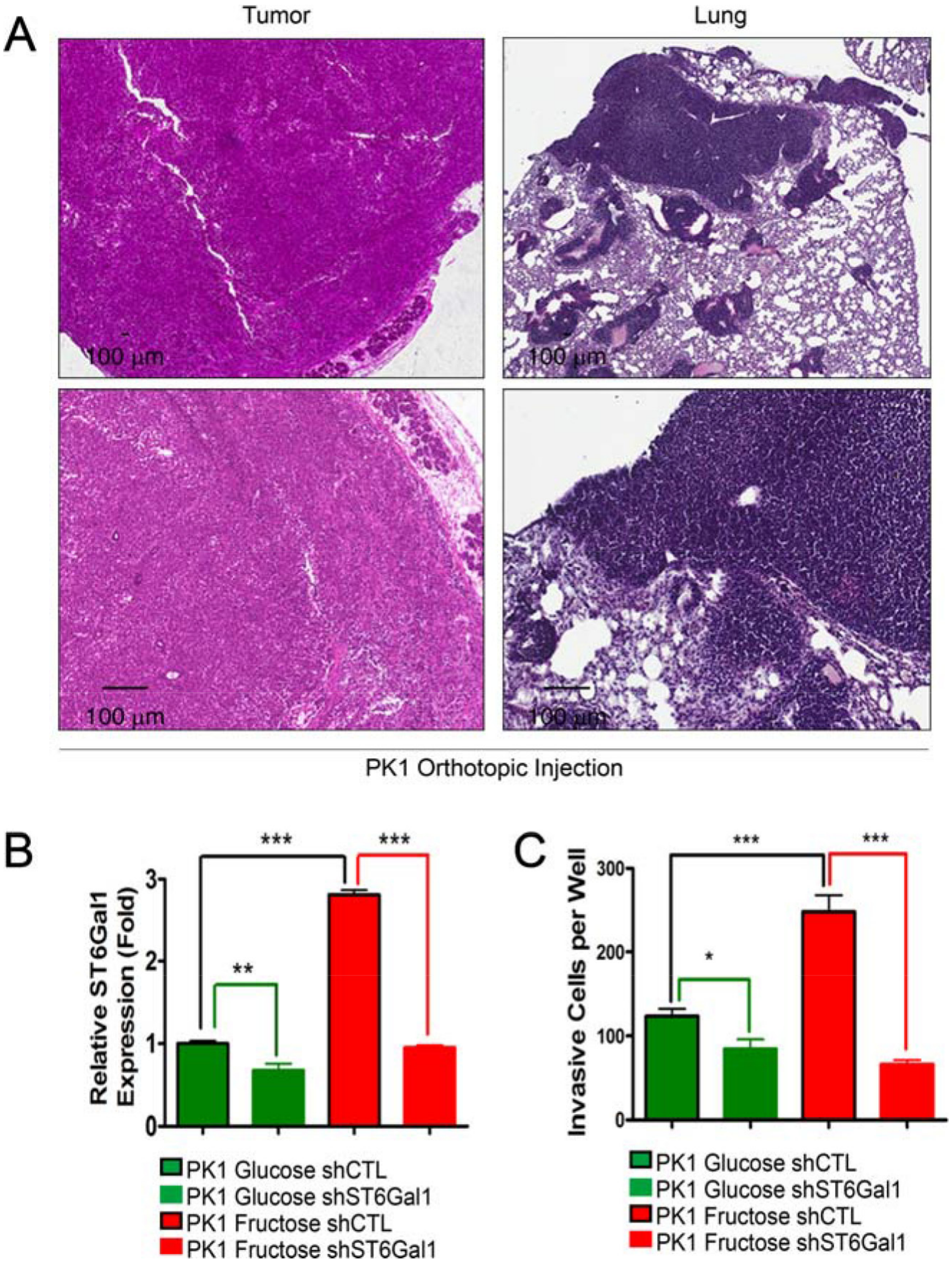

Supplementary Figure S5: ST6Gal1 is also required for invasion triggered by fructose substitution in PK1 cells. (**A**) Pancreatic tumor and lung sections derived from mice injected with PK1 cells were histologically analyzed by H&E staining. Lower panel is magnified to better visualize the morphology of PK1-derived tumor and lung lesions. (**B**–**C**) ST6Gal1 was knockdown in high invasive pancreatic cancer cell PK1 cells. The expression level of *st6gal1* were confirmed by real-time quantitative RT-PCR (B), and compared with that of parental control (glucose shCTL). The invasive capacity conferred by both constructs was verified by matrigel-coated transwell invasion assay (C). Values are shown as the mean ± SEM, $^{*}P < 0.05$, $^{**}P < 0.01$, $^{***}P < 0.001$.

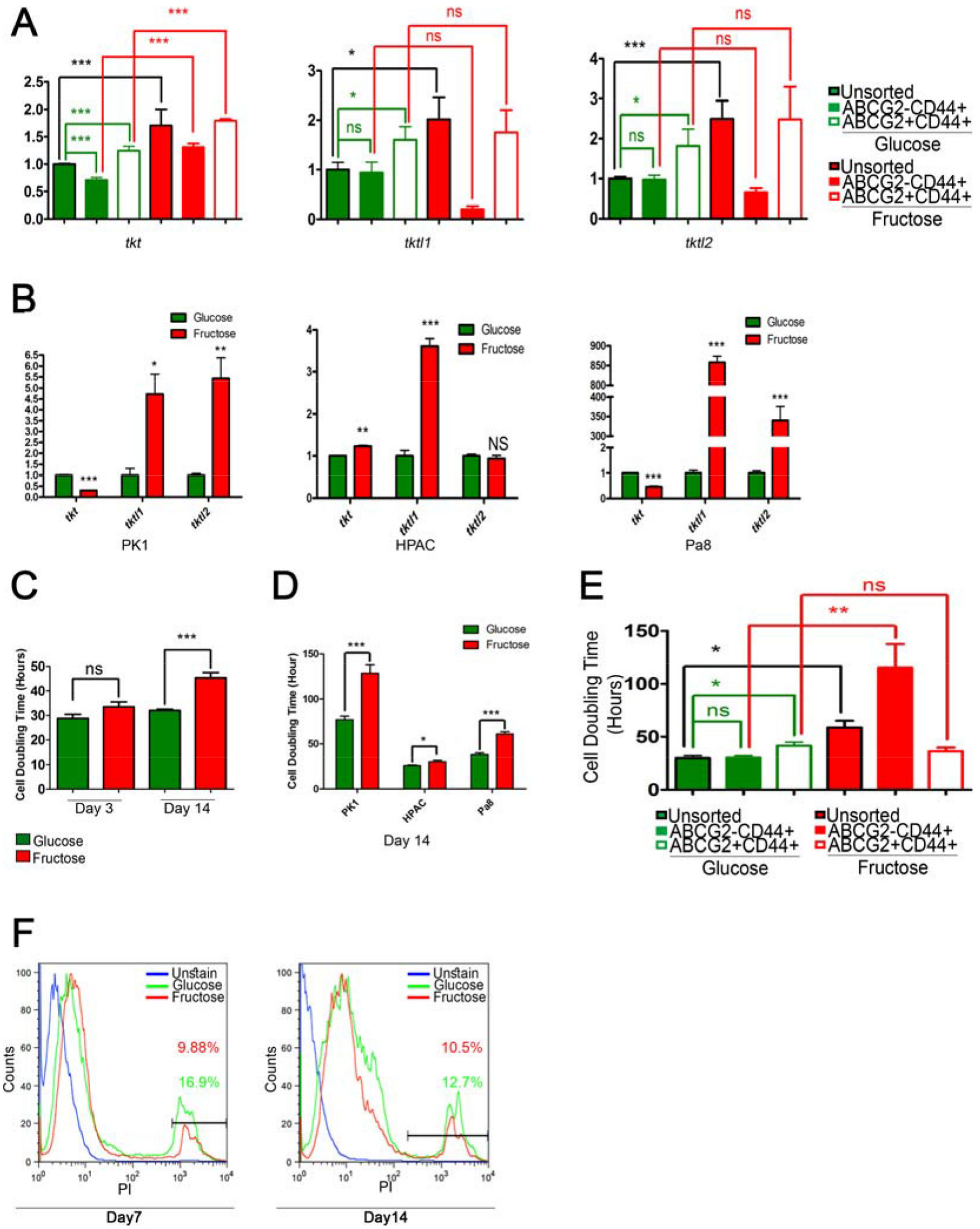

Supplementary Figure S6: Fructose-substituted pancreatic cancer cells exhibit prolonged cell doubling time and higher levels of transketolase. (**A** and **B**) Relative expression profiles of *tkt*, *tktl1*, and *tktl2* in the indicated subpopulations of PANC-1 (A), or PK1, HPAC, and Pa8 cells (B) were examined by real-time quantitative RT-PCR. (**C** and **D**) Cell doubling times of PANC-1 (C), PK1, HPAC, and Pa8 cells (D) passaged in glucose- or in fructose-containing medium were measured by counting cell numbers at the indicated time points. (**E**) Cell doubling time of the indicated subpopulation of parental or 14-day-fructose-substituted PANC-1 cells. (**F**) Representative flow cytometry analysis showing propidium iodide (PI) staining in parental and fructose-substituted PANC-1 at the indicated time points. Blue lines indicate unstained cells. Values are shown as the mean ± SEM, ns indicate non-significant, *$P < 0.05$, **$P < 0.01$, ***$P < 0.001$.

Supplementary Table S1: Subcutaneous tumor formation ability

Conditions	Tumorigenicity	Tumor Doubling Time (Days)	*P*-value
Glucose Unsorted	3/11 (27.27%)	37.42	0.13
Glucose CD44-	1/4 (25.00%)	> 80	0.26
Glucose ABCG2−CD24−CD44+	1/16 (6.25%)	> 80	N/A
Glucose ABCG2−CD24+CD44+	1/4 (25.00%)	27.59	0.26
Glucose ABCG2+CD24−CD44+	2/12 (16.67%)	30.49	0.38
Glucose ABCG2+CD24+CD44+	4/10 (40.00%)	19.40	0.03*

P-value of tumorigenicity is compared with the ABCG2−CD24−CD44+ subpopulation and evaluated by chi-square test. N/A: not available. *$P < 0.05$

Supplementary Table S2: Mutations of cancer cells

Name	Histology	Tumor Source	Mutant Genes	Coding region change	AA mutation
PANC-1	Adenocarcinoma	primary	KRAS<br>TP53<br>TP53	35G > A<br>1020G > A<br>417C > A	Gly12Asp<br>Arg273His<br>Pro72His
PK1	Adenocarcinoma	metastasis, pleural effusion	KRAS<br>TP53	35G > A<br>417C > A	Gly12Asp<br>Pro72His
Pa8	Adenocarcinoma	primary	NRAS<br>TP53	436A > C<br>417C > A	Gln61Pro<br>Pro72His
HPAC	Adenocarcinoma	primary	KRAS<br>TP53<br>KDR	35G > A<br>417C > A<br>1718A > T	Gly12Asp<br>Pro72His<br>Gln472His

Supplementary Table S3: RPKM value of genes of carbohydrate transporter, hexokinase and sialyltransferase identified from PANC-1 cells and ABCG2-positive subpopulations cultured either in glucose-containing medium or fructose substituted medium for 28 days

Carbohydrate transporter and hexokinase

Gene Name	Glucose Unsorted	Glucose ABCG2+	Fructose Unsorted	Glucose Unsorted/Glucose Unsorted (log_2)	Glucose ABCG2+/ Glucose Unsorted (log_2)	Fructose Unsorted/ Glucose Unsorted (log_2)
hk1	34.2545	28.2055	28.8325	0	−0.28032	−0.2486
hk2	2.06062	2.64836	6.02528	0	0.362021	1.54795
hk3	0.02	0.036221	0.02	0	0.85683	0
khk	0.323382	1.25047	0.517083	0	1.951159	0.677156
gck	0.02	0.02	0.02	0	0	0
slc2a1	35.4165	31.8213	106.903	0	−0.15443	1.593809
slc2a2	0.02	0.02	0.02	0	0	0
slc2a3	0.308549	0.458659	0.0670599	0	0.571922	−2.20198
slc2a4	0.260875	0.02	0.262613	0	−3.70529	0.00958
slc2a5	0.02	0.099716	0.243211	0	2.317829	3.604137

Sialyltransferase

Gene Name	Glucose Unsorted	Glucose ABCG2+	Fructose Unsorted	Glucose Unsorted/ Glucose Unsorted (log_2)	Glucose ABCG2+/ Glucose Unsorted (log_2)	Fructose Unsorted/ Glucose Unsorted (log_2)
st3gal1	4.20423	2.46043	4.39758	0	−0.77293	0.064868
st3gal2	25.8332	21.3249	19.6054	0	−0.27669	−0.39798
st3gal3	9.7432	9.68303	9.89526	0	−0.00894	0.022342
st3gal4	28.5539	29.1652	27.8458	0	0.03056	−0.03623
st3gal5	2.30989	3.44434	2.15349	0	0.576403	-0.10115
st3gal6	0.247062	0.486136	0.285982	0	0.976487	0.211051
st6gal1	1.58637	2.14826	5.22662	0	0.437439	1.720149

Supplementary Table S4: Summary of orthotopic pancreatic cancer mice experiments performed in the current study

Cells that were implanted into mice	Related Figure	Age of Mice	Cell Number Injected	Period
28-days Fructose Substituted PANC-1	Figure 2	6–8 weeks	1×10^4	16 weeks
ABCG2-positive Subpopulation of PANC-1 with ST6Gal1 Overexpression	Figure 6	6–8 weeks	5×10^3	14 weeks
ABCG2-positive Subpopulation of 28-days Fructose Substituted PANC-1 with ST6Gal1 Knockdown	Figure 7	6–8 weeks	5×10^3	14 weeks

Supplementary Table S5: Correlation of ST6Gal1 expression and clinical outcome of 51 patients with PDAC

	ST6Gal1		
	Low (n = 31)	High (n = 20)	P-Value
Gender			
Male (n = 32)	23 (74)	9 (45)	
Female (n = 19)	8 (26)	11 (55)	0.03*
Age	65 ± 12	71 ± 13	0.112
Serum tumor marker			
CEA	40 ± 131	60 ± 161	0.656
CA19-9	915 ± 2055	376 ± 1223	0.308
Tumor size (cm)	2.62 ± 0.7	3.22 ± 1.3	0.038*
Lymph node metastasis, yes	17 (55)	13 (65)	0.472
Tumor–node–metastasis stage			
I	2 (6)	0 (0)	
II	24 (77)	18 (90)	
III	3 (10)	1 (5)	
IV	2 (6)	1 (5)	0.592

Values in parentheses indicate percentages.

Supplementary Table S6: List of Primers used for qPCR

Gene	Forward	Reverse
gapdh	AGCCACATCGCTCAGACAC	GCCCAATACGACCAAATCC
abcg2	AGTTCCATGGCACTGGCCATA	TCAGGTAGGCAATTGTGAGG
hk1	GCTCTCCGATGAAACTCTCATAG	GGACCTTACGAATGTTGGCAA
hk2	TCCCCTGCCACCAGACTA	TGGACTTGAATCCCTTGGTC
slc2a1	TCTGGCATCAACGCTGTCTTC	CGATACCGGAGCCAATGGT
slc2a2	GCCTGGTTCCTATGTATATCGGT	GCCACAGATCATAATTGCCCAAG
slc2a3	GCTGGGCATCGTTGTTGGA	GCACTTTGTAGGATAGCAGGAAG
slc2a4	TGGGCGGCATGATTTCCTC	GCCAGGACATTGTTGACCAG
slc2a5	CGTGCCTGCGATCTTAATGG	GATACACCTGCACATATTCCCAC
st3gal1	CAAATCCCGGAAACTCCAG	TTCCTCTCATTGACATTTCCAG
st3gal2	GTCCAGAGGTGGTGGATGAT	CAGCACCTCATTGGTGTTGT
st3gal3	GCACCCCTGCACTACTATGAG	GCTGGATATTGTGCGTCCA
st3gal4	TGAATCTGCCCACTTCGACC	CTTGAAAGCTACCAGGACGAG
st3gal5	TATAGCGTGGACTTACTCCCTTT	AGGAGGATCGTACTTGGACTC
st3gal6	CCAGCCTTGTTTATCAAAGCCA	AGGGCAAATCAAACTTATCGCTA
st6gal1	ACCCCAATCAGCCCTTTTACA	CTGGTCACACAGCGTCATCA
tkt	CGGCAAATACTTCGACAAGG	ATGGCCTCCCATACAGAGC
tktl1	CCACCTGATTACAGAGTTGGTG	CTCTGTTGTTCGCGTAGCC
tktl2	ACGACCGGTTCATCCTCTC	TCCACCCAAGCAGCATAGA

Supplementary Table S7: ST6Gal1 shRNA sequence information

**Target Gene**	ST6Gal1
**Vector**	pLKO.1
**Target Sequence**	CCCAGAAGAGATTCAGCCAAA
**Oligo Sequence**	CCGGCGTGTGCTACTACTACCAGAACTCGAGTTCTGGTAGTAGTAGCACACGTTTTTG
