# Supplementary material for: Elevation of β-galactoside α2,6-sialyltransferase 1 in a fructose-responsive manner promotes pancreatic cancer metastasis
Source: Oncotarget. 2016 Dec 9;8(5):7691–709. doi: 10.18632/oncotarget.13845 (PMC5352353; doi:10.18632/oncotarget.13845)
Supplement: Supplementary file 2 [file oncotarget-08-7691-s002.docx]

**Supplementary Table S1: Subcutaneous tumor formation ability**

| **Conditions** | **Tumorigenicity** | **Tumor Doubling Time (Days)** | ***P*-value** |
| --- | --- | --- | --- |
| Glucose Unsorted | 3/11 (27.27%) | 37.42 | 0.13 |
| Glucose CD44- | 1/4 (25.00%) | > 80 | 0.26 |
| Glucose ABCG2−CD24−CD44+ | 1/16 (6.25%) | > 80 | N/A |
| Glucose ABCG2−CD24+CD44+ | 1/4 (25.00%) | 27.59 | 0.26 |
| Glucose ABCG2+CD24−CD44+ | 2/12 (16.67%) | 30.49 | 0.38 |
| Glucose ABCG2+CD24+CD44+ | 4/10 (40.00%) | 19.40 | 0.03^*^ |
| *P*-value of tumorigenicity is compared with the ABCG2−CD24−CD44+ subpopulation and evaluated by chi-square test. N/A: not available. ^*^*P* < 0.05 | | | |

**Supplementary Table S2: Mutations of cancer cells**

| **Name** | **Histology** | **Tumor Source** | **Mutant Genes** | **Coding region change** | **AA mutation** |
| --- | --- | --- | --- | --- | --- |
| PANC-1 | Adenocarcinoma | primary | KRAS  TP53  TP53 | 35G > A  1020G > A  417C > A | Gly12Asp  Arg273His  Pro72His |
| PK1 | Adenocarcinoma | metastasis,  pleural effusion | KRAS  TP53 | 35G > A  417C > A | Gly12Asp  Pro72His |
| Pa8 | Adenocarcinoma | primary | NRAS  TP53 | 436A > C  417C > A | Gln61Pro  Pro72His |
| HPAC | Adenocarcinoma | primary | KRAS  TP53  KDR | 35G > A  417C > A  1718A > T | Gly12Asp  Pro72His  Gln472His |

**Supplementary Table S3: RPKM value of genes of carbohydrate transporter, hexokinase and sialyltransferase identified from PANC-1 cells and ABCG2-positive subpopulations cultured either in glucose-containing medium or fructose substituted medium for 28 days**

| **Carbohydrate transporter and hexokinase** | | | | | | |
| --- | --- | --- | --- | --- | --- | --- |
| **Gene Name** | **Glucose**  **Unsorted** | **Glucose ABCG2+** | **Fructose Unsorted** | **Glucose**  **Unsorted/Glucose**  **Unsorted (log_2_)** | **Glucose ABCG2+/Glucose Unsorted (log_2_)** | **Fructose Unsorted/ Glucose Unsorted (log_2_)** |
| *hk1* | 34.2545 | 28.2055 | 28.8325 | 0 | −0.28032 | −0.2486 |
| *hk2* | 2.06062 | 2.64836 | 6.02528 | 0 | 0.362021 | 1.54795 |
| *hk3* | 0.02 | 0.036221 | 0.02 | 0 | 0.85683 | 0 |
| *khk* | 0.323382 | 1.25047 | 0.517083 | 0 | 1.951159 | 0.677156 |
| *gck* | 0.02 | 0.02 | 0.02 | 0 | 0 | 0 |
| *slc2a1* | 35.4165 | 31.8213 | 106.903 | 0 | −0.15443 | 1.593809 |
| *slc2a2* | 0.02 | 0.02 | 0.02 | 0 | 0 | 0 |
| *slc2a3* | 0.308549 | 0.458659 | 0.0670599 | 0 | 0.571922 | −2.20198 |
| *slc2a4* | 0.260875 | 0.02 | 0.262613 | 0 | −3.70529 | 0.00958 |
| *slc2a5* | 0.02 | 0.099716 | 0.243211 | 0 | 2.317829 | 3.604137 |
| **Sialyltransferase** | | | | | | |
| **Gene Name** | **Glucose**  **Unsorted** | **Glucose ABCG2+** | **Fructose Unsorted** | **Glucose**  **Unsorted/ Glucose**  **Unsorted (log_2_)** | **Glucose ABCG2+/ Glucose**  **Unsorted (log_2_)** | **Fructose Unsorted/ Glucose**  **Unsorted (log_2_)** |
| *st3gal1* | 4.20423 | 2.46043 | 4.39758 | 0 | −0.77293 | 0.064868 |
| *st3gal2* | 25.8332 | 21.3249 | 19.6054 | 0 | −0.27669 | −0.39798 |
| *st3gal3* | 9.7432 | 9.68303 | 9.89526 | 0 | −0.00894 | 0.022342 |
| *st3gal4* | 28.5539 | 29.1652 | 27.8458 | 0 | 0.03056 | −0.03623 |
| *st3gal5* | 2.30989 | 3.44434 | 2.15349 | 0 | 0.576403 | -0.10115 |
| *st3gal6* | 0.247062 | 0.486136 | 0.285982 | 0 | 0.976487 | 0.211051 |
| *st6gal1* | 1.58637 | 2.14826 | 5.22662 | 0 | 0.437439 | 1.720149 |

**Supplementary Table S4: Summary of orthotopic pancreatic cancer mice experiments performed in the current study**

| **Cells that were implanted into mice** | **Related Figure** | **Age of Mice** | **Cell Number Injected** | **Period** |
| --- | --- | --- | --- | --- |
| 28-days Fructose Substituted PANC-1 | Figure 2 | 6–8 weeks | 1 × 10^4^ | 16 weeks |
| ABCG2-positive Subpopulation of PANC-1 with ST6Gal1 Overexpression | Figure 6 | 6–8 weeks | 5 × 10^3^ | 14 weeks |
| ABCG2-positive Subpopulation of 28-days Fructose Substituted PANC-1 with ST6Gal1 Knockdown | Figure 7 | 6–8 weeks | 5 × 10^3^ | 14 weeks |

**Supplementary Table S5: Correlation of ST6Gal1 expression and clinical outcome of 51 patients with PDAC**

|  | **ST6Gal1** | |  |
| --- | --- | --- | --- |
|  | **Low (*n* = 31)** | **High (*n* = 20)** | ***P*-Value** |
| Gender |  |  |  |
| Male (*n* = 32) | 23 (74) | 9 (45) |  |
| Female (*n* = 19) | 8 (26) | 11 (55) | 0.03^*^ |
| Age | 65 ± 12 | 71 ± 13 | 0.112 |
| Serum tumor marker |  |  |  |
| CEA | 40 ± 131 | 60 ± 161 | 0.656 |
| CA19-9 | 915 ± 2055 | 376 ± 1223 | 0.308 |
| Tumor size (cm) | 2.62 ± 0.7 | 3.22 ± 1.3 | 0.038^*^ |
| Lymph node metastasis, yes | 17 (55) | 13 (65) | 0.472 |
| Tumor–node–metastasis stage |  |  |  |
| I | 2 (6) | 0 (0) |  |
| II | 24 (77) | 18 (90) |  |
| III | 3 (10) | 1 (5) |  |
| IV | 2 (6) | 1 (5) | 0.592 |
| Values in parentheses indicate percentages. |  |  |  |

**Supplementary Table S6: List of Primers used for qPCR**

| **Gene** | **Forward** | **Reverse** |
| --- | --- | --- |
| *gapdh* | AGCCACATCGCTCAGACAC | GCCCAATACGACCAAATCC |
| *abcg2* | AGTTCCATGGCACTGGCCATA | TCAGGTAGGCAATTGTGAGG |
| *hk1* | GCTCTCCGATGAAACTCTCATAG | GGACCTTACGAATGTTGGCAA |
| *hk2* | TCCCCTGCCACCAGACTA | TGGACTTGAATCCCTTGGTC |
| *slc2a1* | TCTGGCATCAACGCTGTCTTC | CGATACCGGAGCCAATGGT |
| *slc2a2* | GCCTGGTTCCTATGTATATCGGT | GCCACAGATCATAATTGCCCAAG |
| *slc2a3* | GCTGGGCATCGTTGTTGGA | GCACTTTGTAGGATAGCAGGAAG |
| *slc2a4* | TGGGCGGCATGATTTCCTC | GCCAGGACATTGTTGACCAG |
| *slc2a5* | CGTGCCTGCGATCTTAATGG | GATACACCTGCACATATTCCCAC |
| *st3gal1* | CAAATCCCGGAAACTCCAG | TTCCTCTCATTGACATTTCCAG |
| *st3gal2* | GTCCAGAGGTGGTGGATGAT | CAGCACCTCATTGGTGTTGT |
| *st3gal3* | GCACCCCTGCACTACTATGAG | GCTGGATATTGTGCGTCCA |
| *st3gal4* | TGAATCTGCCCACTTCGACC | CTTGAAAGCTACCAGGACGAG |
| *st3gal5* | TATAGCGTGGACTTACTCCCTTT | AGGAGGATCGTACTTGGACTC |
| *st3gal6* | CCAGCCTTGTTTATCAAAGCCA | AGGGCAAATCAAACTTATCGCTA |
| *st6gal1* | ACCCCAATCAGCCCTTTTACA | CTGGTCACACAGCGTCATCA |
| *tkt* | CGGCAAATACTTCGACAAGG | ATGGCCTCCCATACAGAGC |
| *tktl1* | CCACCTGATTACAGAGTTGGTG | CTCTGTTGTTCGCGTAGCC |
| *tktl2* | ACGACCGGTTCATCCTCTC | TCCACCCAAGCAGCATAGA |

**Supplementary Table S7: ST6Gal1 shRNA sequence information**

| **Target Gene** | ST6Gal1 |
| --- | --- |
| **Vector** | pLKO.1 |
| **Target Sequence** | CCCAGAAGAGATTCAGCCAAA |
| **Oligo Sequence** | CCGGCGTGTGCTACTACTACCAGAACTCGAGTTCTGGTAGTAGTAGCACACGTTTTTG |
